# Supplementary figures and images for: A sensitive soma-localized red fluorescent calcium indicator for in vivo imaging of neuronal populations at single-cell resolution
Source: PLoS Biol. 2025 Apr 29;23(4):e3003048. doi: 10.1371/journal.pbio.3003048 (PMC12040222; doi:10.1371/journal.pbio.3003048)

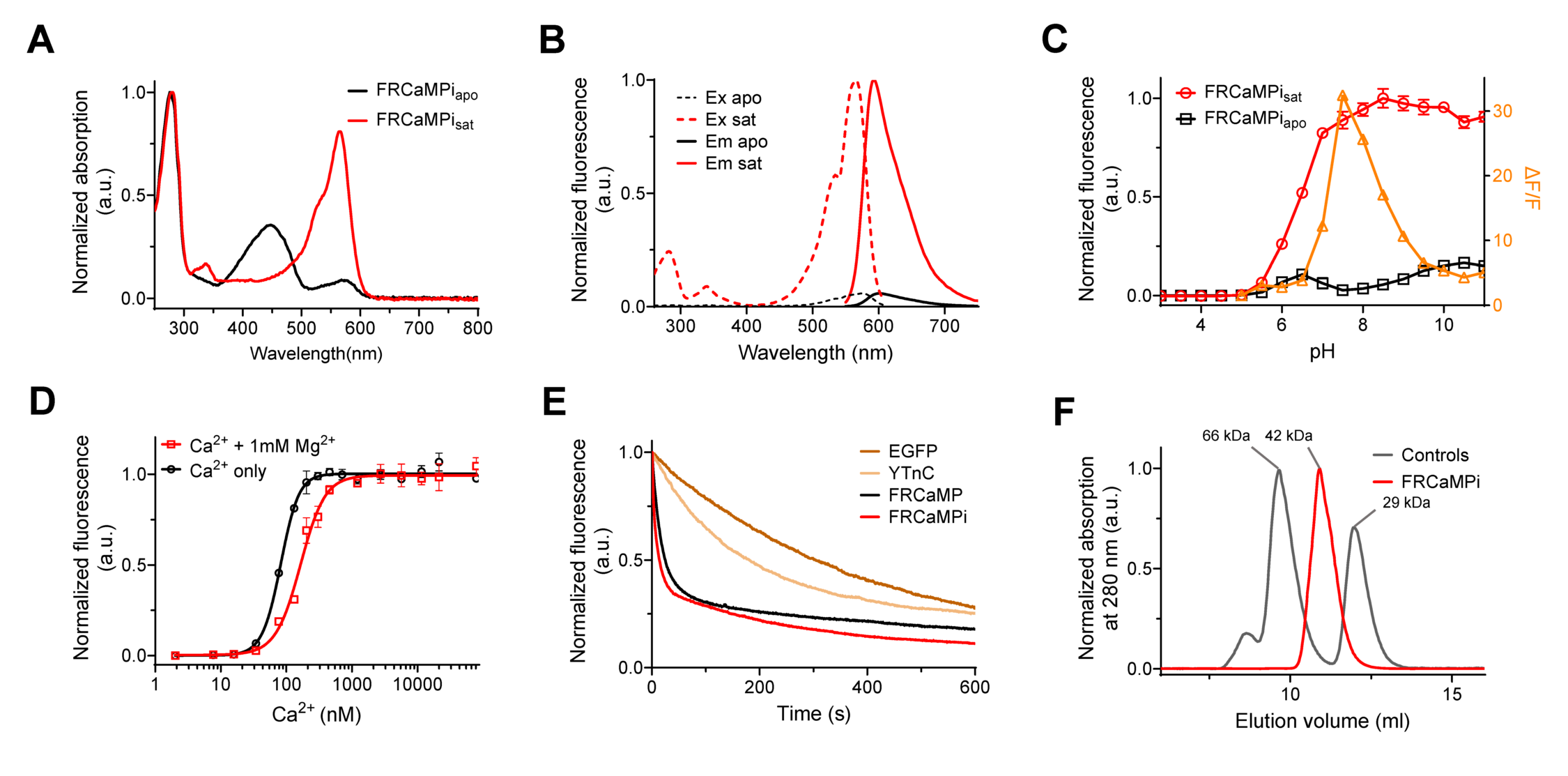

Supplement: S1 Fig — (A) Absorption spectra for FRCaMPi in the Ca2+-bound (sat) and Ca2+-free (apo) states at pH 7.20. (B) Excitation and emission spectra for FRCaMPi in the Ca2+-bound (sat) and Ca2+-free (apo) states at pH 7.20. (C) Red fluorescence intensity for FRCaMPi in the Ca2+-bound (sat) and Ca2+-free (sat) states and the F/F dynamic range as a function of pH. (D) Ca2+ titration curves for FRCaMPi in the absence and presence of 1 mM MgCl2 at pH 7.20. The experimental data were fitted by the Hill equation. (E) Photobleaching of FRCaMPi and control FRCaMP, YTnC, and mEGFP proteins under continuous wide-field imaging using a mercury lamp in the presence of Ca2+. (F) Fast protein liquid chromatography of FRCaMPi in the presence of Ca2+. FRCaMPi (4.5 mg/ml) was eluted in 40 mM Tris-HCl (pH 7.5) and 200 mM NaCl buffer supplemented with 5 mM CaCl2. The molecular weight of FRCaMPi (having a theoretical molecular weight of 48 kDa) was calculated from a linear regression of the dependence of the logarithm of the control molecular weights versus the elution volume. (C–F) Three–six replicates were averaged for analysis. Error bars represent the standard deviation. (TIF) [file pbio.3003048.s001.tif]

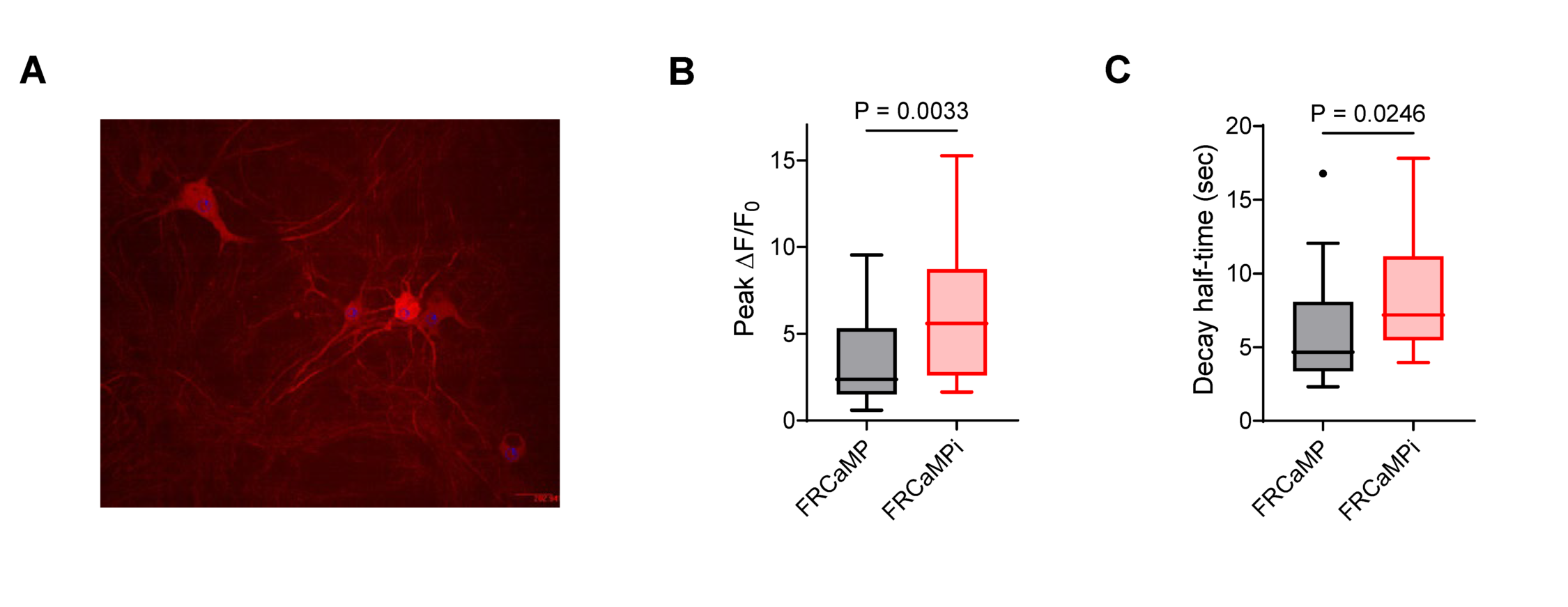

Supplement: S2 Fig — (A) Representative images from neuronal culture expressing FRCaMPi. (B) Peak ΔF/F0 of FRCaMP and FRCaMPi at 87 Hz, 300 pulses. (C) Half decay time of FRCaMP and FRCaMPi at 87 Hz, 300 pulses (25 cells each, from 2 independent cultures). Mann–Whitney U test: not significant. Box indicates the median and 25–75th percentile range, and the whiskers represent 1.5 times of interquartile range. The quantitative data presented in this figure can be found in S2 Data. (TIF) [file pbio.3003048.s002.tif]

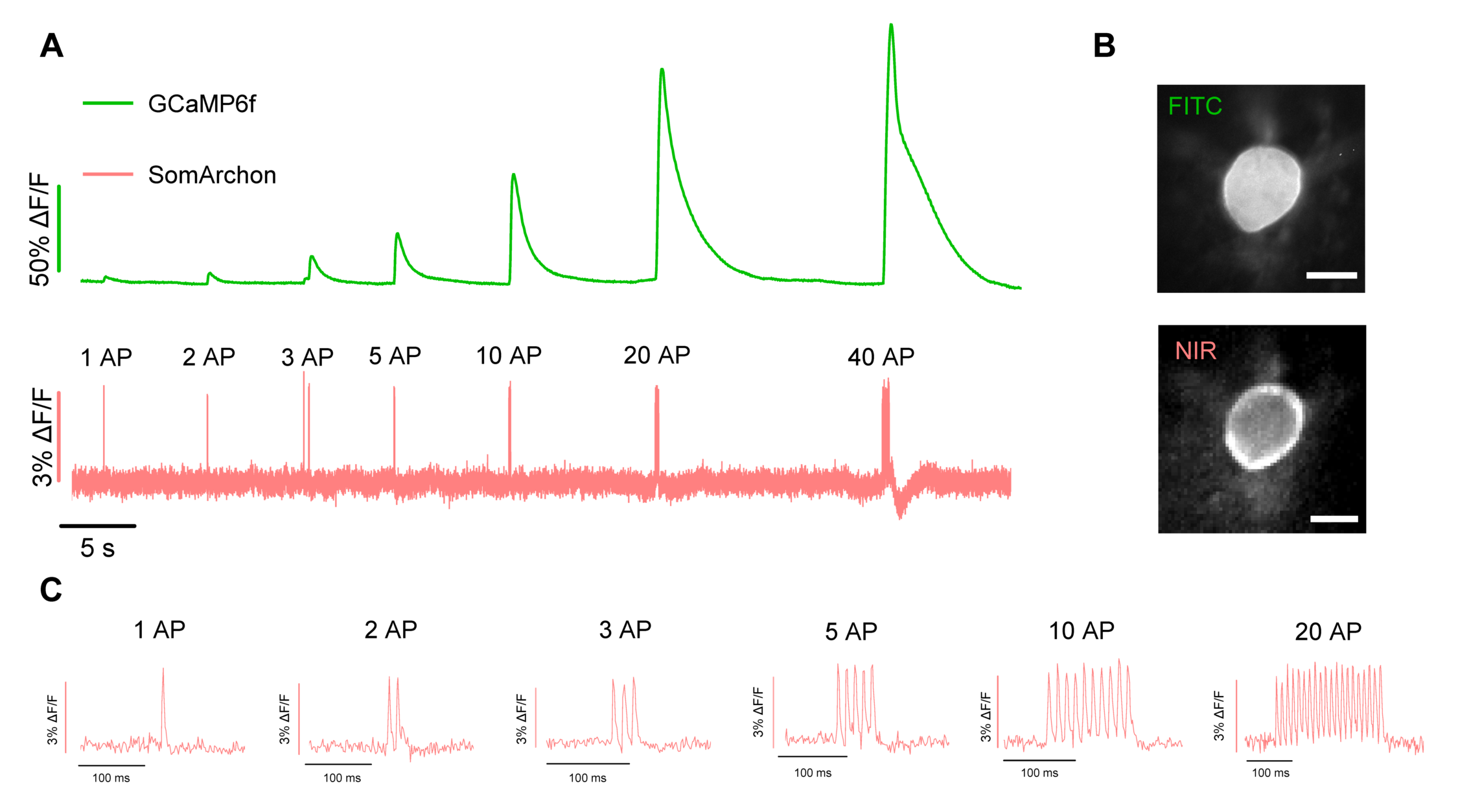

Supplement: S3 Fig — (A) Representative trace of concurrent voltage and calcium recording in neuron co-expressing GCaMP6f and SomArchon. Pulse numbers 1, 2, 3, 5, 10, 20, and 40 were evoked with 1 ms width and frequency of 83 Hz. 40–50 V voltage was applied. An extra spike was detected before 3 AP stimulation. Images were sampled at 1 kHz for NIR and 25 Hz for FITC channel. (B) Representative standard deviation projection image of neuron from (A) showing the maximal fluorescence across all the frames. (C) Cropped voltage traces showing the exact spike number from voltage recording in (A). The numbers of external electrical pulses applied were aligned with the number of spikes observed, indicated by fluorescence of SomArchon. (TIF) [file pbio.3003048.s003.tif]

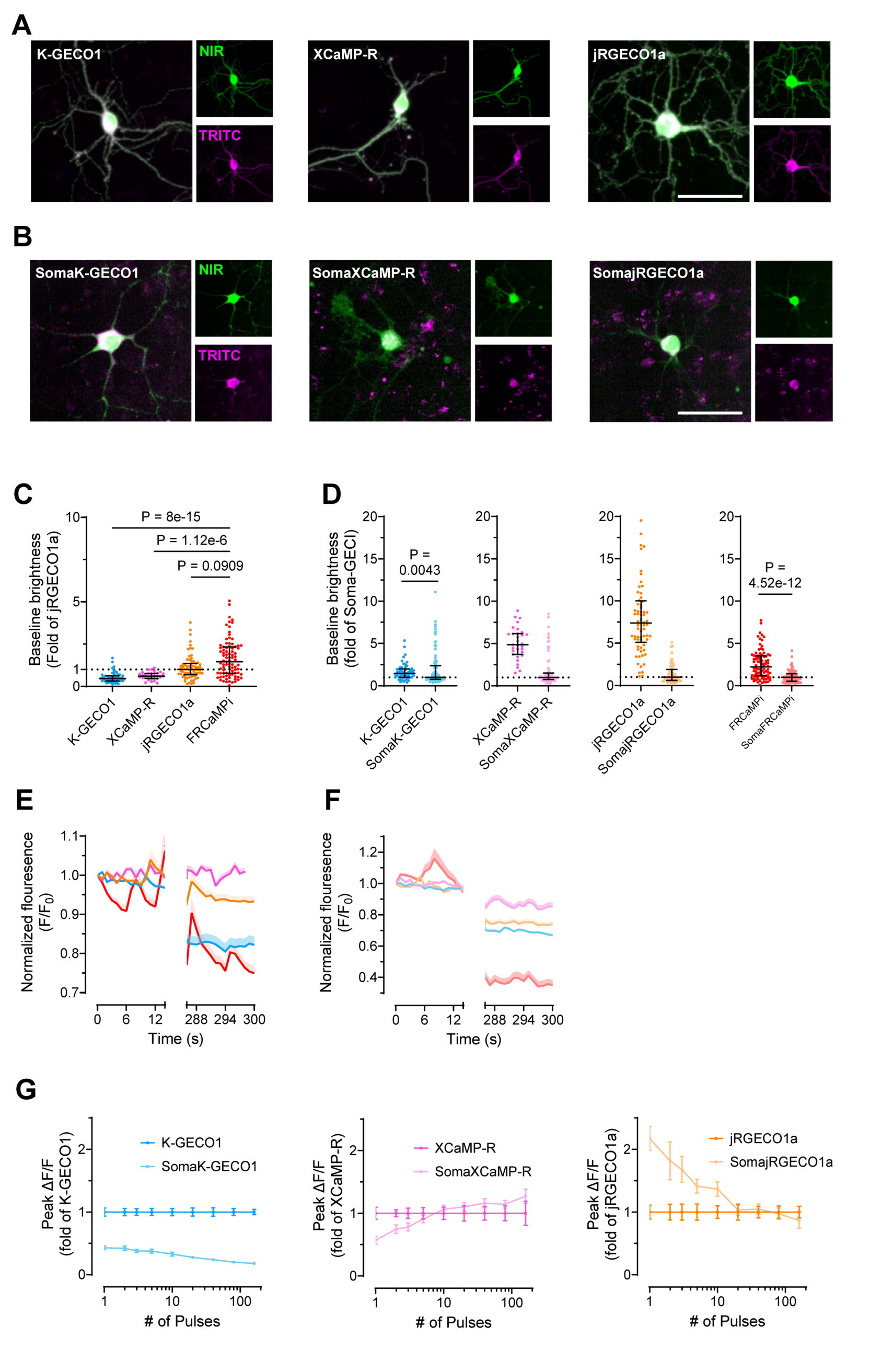

Supplement: S4 Fig — (A) Representative images of neuron co-expressing red GECI (TRITC) and emiRFP670 (NIR). (n = 5 from two independent neuronal cultures). (B) Representative images of neuron co-expressing soma-localized version of red GECI (TRITC) and emiRFP670 (NIR) (top). Scale bar, 50 µm. (n = 5 from two independent neuronal cultures). (C) Baseline brightness of red GECIs characterized in transduced primary hippocampal neurons by side-by-side comparison. K-GECO1: n = 55 cells from 3 wells; XCaMP-R: n = 31 cells from 2 wells; jRGECO1a: n = 59 cells from 3 wells; FRCaMPi: n = 87 cells from 4 wells, two independent cultures. Kruskal–Wallis multiple-comparison test. Data is normalized to the median of the jRGECO1a values. (D) Baseline brightness of red GECIs compared to that of their soma-localized version. Data is normalized to the median of the soma-localized GECI values. Statistics in d for are the same as in c and Fig 2H. (E) 5 min recording of fluorescence trace from neurons expressing red GECI before applying electrical field stimuli. n = 34 cells for K-GECO1, n = 31 cells for XCaMPR, n = 49 cells for jRGECO1a, n = 14 cells for FRCaMPi. (F) As in f[To AU: Please check whether the part label “f” referred here is correct. Please confirm.] but for soma-localized red GECIs. n = 35 cells for SomaK-GECO1, n = 51 cells for SomaXCaMP-R, n = 59 cells for SomajRGECO1a, n = 28 cells for SomaFRCaMPi. (G) Peak ΔF/F0 of red GECI compared to that of their corresponding soma-localized version as a function of pulses K-GECO1: n = 54 cells from 3 coverslips, 2 independent cultures; SomaK-GECO1: n = 61 cells from 4 coverslips, 3 independent cultures; XCaMP-R: n = 24 cells from 2 coverslips, 2 independent cultures; SomaXCaMP-R: n = 64 cells from 4 coverslips, 3 independent cultures; jRGECO1a: n = 65 cells from 4 coverslips, 3 independent cultures; SomajRGECO1a: n = 100 cells from 6 coverslips, 4 independent cultures. The quantitative data presented in this figure can be found in S2 Data. (TIF) [file pbio.3003048.s004.tif]

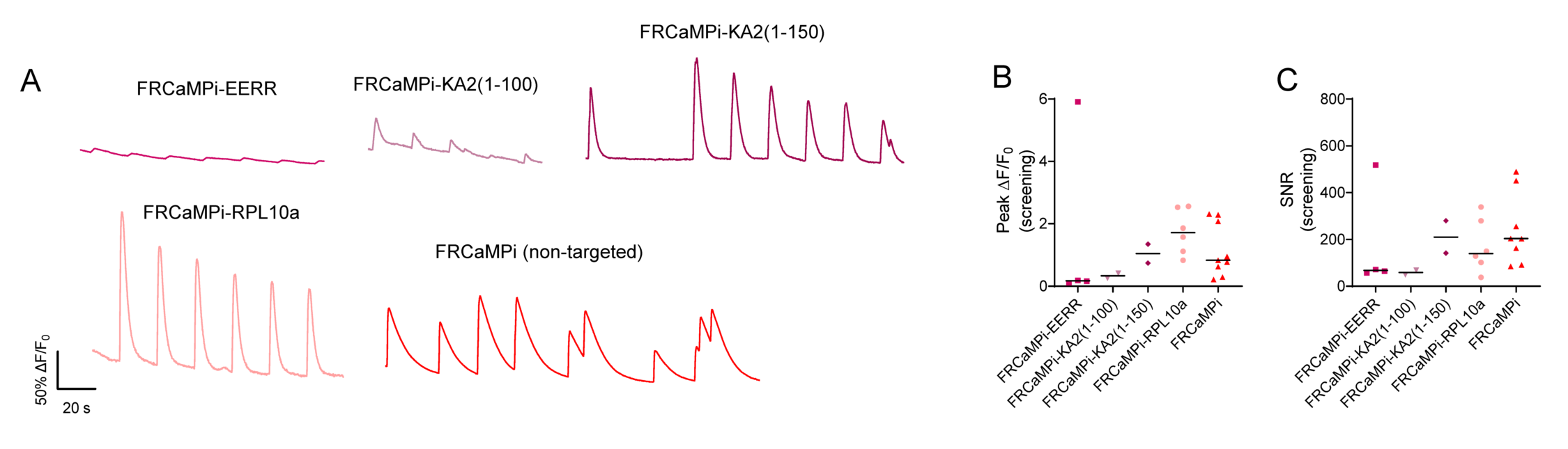

Supplement: S6 Fig — We made fusions between FRCaMPi and the peptides known to facilitate somatic localization. We then screened those fusions with a focus on their functionality: cytotoxicity (assessed as total number of functional cells obtained from transfected neuronal culture), (A) single-trial single-cell optical traces from representative neurons expressing (B) fluorescence changes (peak/max ΔF/F0) and (C) SNR in response to evoked extracellular electrical stimuli (n = X, Y, Z neurons from 1 matching culture for, respectively). We found that ribo-tagged FRCaMPi (FRCaMPi-RPL10a) demonstrates the least toxicity, highest peak ΔF/F0 on average with SNR comparable to FRCaMPi. We thereby pursued the ribo-tagged construct for more detailed characterization, naming it SomaFRCaMPi. The quantitative data presented in this figure can be found in S2 Data. (TIF) [file pbio.3003048.s006.tif]

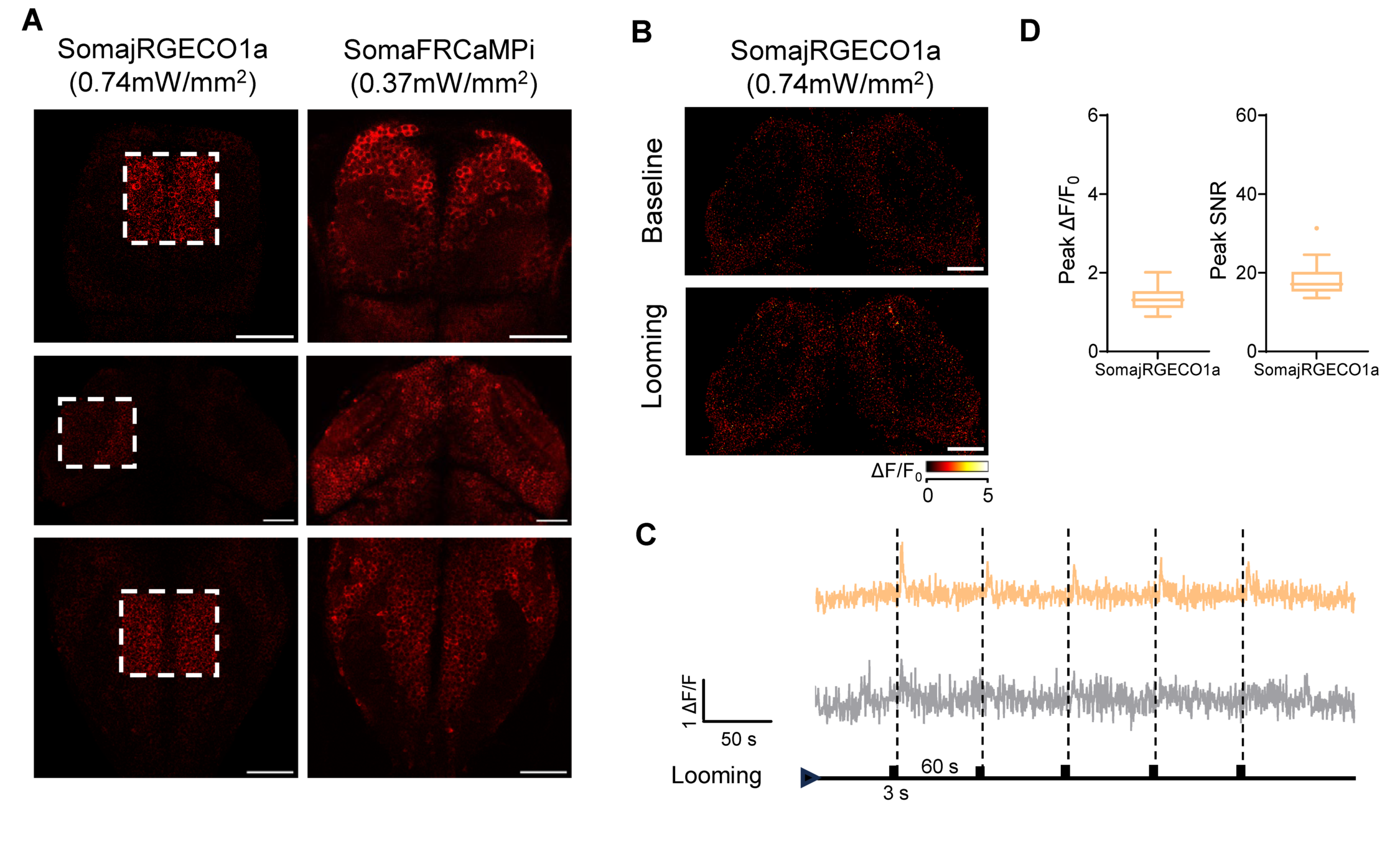

Supplement: S7 Fig — (A) In vivo confocal images showing fore-, mid- and hindbrain region of live F1 zebrafish larva expressing SomajRGECO1a and SomaFRCaMPi in neurons driven by the HuC promoter. The dynamic range was adjusted identical for each image to facilitate visual comparison of brightness. SomajRGECO1a was imaged at 2 mW whereas SomaFRCaMPi was imaged using 0.8 mW laser power. The white dashed box indicates local area of SomajRGECO1a image adapted enhanced contrast to aid visualization. The baseline brightness of SomajRGECO1a in F1 is approximately three times dimmer compared to that of SomaFRCaMPi. (B) Representative confocal images showing fluorescence response of optical tectum in larva expressing SomajRGECO1a before and during looming stimulation periods. The laser power used to image SomajRGECO1a in F1 fish (0.74 mW/mm2) was 10 times to that of jRGECO1a, FRCaMPi and SomaFRCaMPi in F3 (0.076 mW/mm2). (C) Representative fluorescence traces of soma and neuropil region in optical tectum of larva expressing SomajRGECO1a during looming stimulation. (D) Peak ΔF/F0 and peak SNR of the fluorescence response at neuronal soma of SomajRGECO1a-expressing fish during looming stimulation. n = 24 cells from 4 fish. Scale bar, 50 μm. (TIF) [file pbio.3003048.s007.tif]

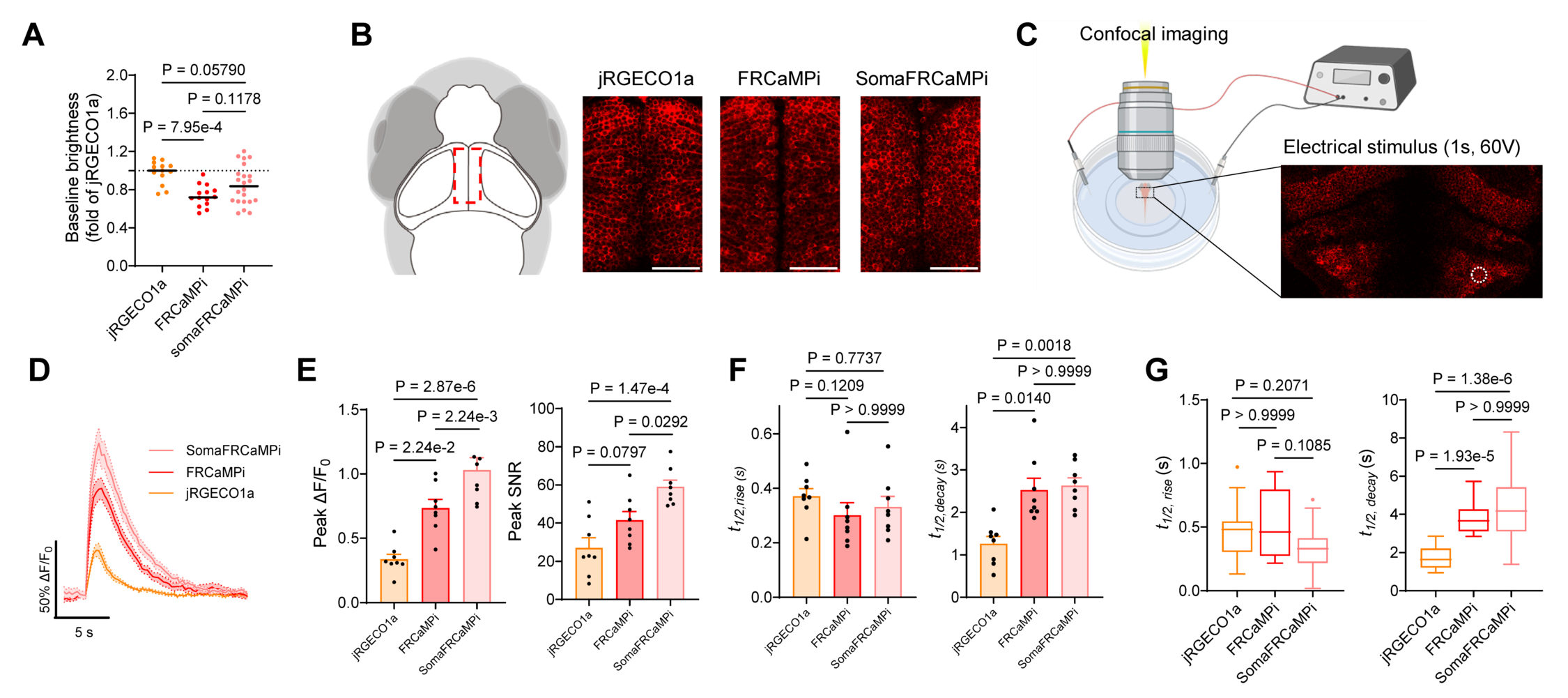

Supplement: S8 Fig — (A) Baseline brightness of larval fish expressing jRGECO1a or FRCaMPi variants n = 13 fish for jRGECO1a, n = 14 fish for FRCaMPi, n = 22 fish for SomaFRCaMPi. c. Half rise (left) and decay (right) time of red GECIs in response to looming stimuli. (B) Representative single plane confocal images of periventricular cell bodies in optic tectum of fish expressing jRGECO1a, FRCaMPi or SomaFRCaMPi. Decreased neuropil fluorescence and clearer neuronal cell bodies was found in SomaFRCaMPi-expressing larval zebrafish. (C) Larval zebrafish were mounted into agarose gel and calcium response was evoked by electrical stimuli delivered by platinum sheet electrodes and a voltage amplifier. Neurons activity in the superior medulla oblongata were recorded. (D) Averaged ΔF/F0 Ca2+ transients from neurons in the superior medulla oblongata, responding to electrical field stimulation. Averaged traces are shown as mean ± s.e.m, indicated by solid lines and shaded area, respectively. (E) Peak ΔF/F0 (left) and peak SNR (right) of neurons expressing jRGECO1a, FRCaMPi or SomaFRCaMPi in the superior medulla oblongata, responding to electrical field stimulation. One-way ANOVA test was used followed by a post hoc Tukey test. The Shapiro–Wilk test was used for the normal distribution test. (F) Half rise (left) and decay (right) time of neurons expressing jRGECO1a or FRCaMPi variants in the superior medulla oblongata, responding to electrical field stimulation. A single circular ROI containing 4–5 neurons was selected for each fish. A total of 8 fish were recorded for jRGECO1a, FRCaMPi and SomaFRCaMPi each. Statistics in d–e are the same as in f. (G) Half rise (left) and decay (right) time of the fluorescence response during looming stimulation (jRGECO1a: n = 18 cells; FRCaMPi: n = 18 cells; SomaFRCaMPi: n = 17, 3 fish each) Kruskal–Wallis ANOVA was used followed by a post hoc Dunn’s multiple comparison test. In dot plots, dots are individual data points; the line denotes the median. Bar chart is [file pbio.3003048.s008.tif]

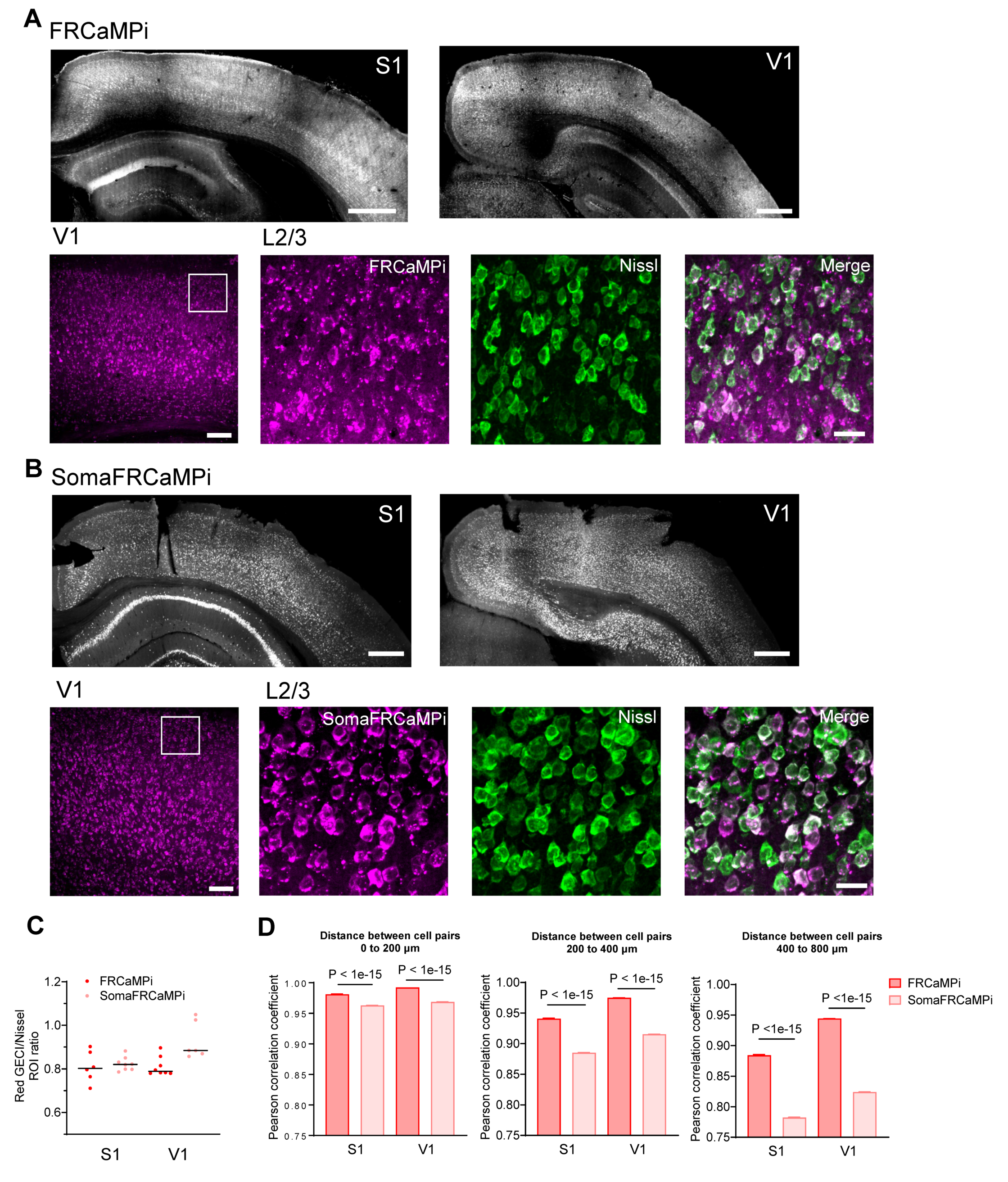

Supplement: S11 Fig — (A) Top: coronal section demonstrating that FRCaMPi are expressed pan-cortically in S1 (left) and V1 (right) region. Bottom: representative image showing L2/3 neurons in V1 with FRCaMPi (magenta) and Nissl staining (green). (B) Same as a but for a mouse brain expressing SomaFRCaMPi. Adeno-associated viruses encoding FRCaMPi or SomaFRCaMPi were delivered by intracerebral ventricular injection into neonatal mice bilaterally. (C) Ratio of FRCaMPi- or SomaFRCaMPi-expressing neurons to Nissl positive neurons quantifies the labeling density at region S1 or V1. S1: n = 6 slides from 2 FRCaMPi mice, n = 8 slides from 3 SomaFRCaMPi mice; V1: n = 8 slides from 3 FRCaMPi mice, n = 6 slides from 2 SomaFRCaMPi mice. (D) Pearson correlation coefficient of calcium dynamics between pairs of neurons within the range of 0–200 μm, 200–400 μm and 400–800 μm. Statistics is the same as in Figs 5K and l. Mann–Whitney U test was used. Scale bar, 25 μm, 100 μm and 500 μm. Bar chart is shown as mean ± s.e.m. Box indicates the median and 25–75th percentile range, and the whiskers represent 1.5 times of interquartile range. Pearson correlation coefficient is plotted as bar to aid demonstration of data distribution. See S6 Table for more details. The quantitative data presented in this figure can be found in S2 Data. (TIF) [file pbio.3003048.s011.tif]
