# Supplementary material for: A sensitive soma-localized red fluorescent calcium indicator for in vivo imaging of neuronal populations at single-cell resolution
Source: PLoS Biol. 2025 Apr 29;23(4):e3003048. doi: 10.1371/journal.pbio.3003048 (PMC12040222; doi:10.1371/journal.pbio.3003048)
Supplement: S1 Table — (DOCX) [file pbio.3003048.s017.docx]

**S1 Table.** **List of Amino acid sequences for peptide screening**

**Linker:**

GGSGGSGGTGGSGGSGGTGGSGGSGGT

**KA2(1-150):**

MPAELLLLLIVAFANPSCQVLSSLRMAAILDDQTVCGRGERLALALAREQINGIIEVPAKARVEVDIFELQRDSQYETTDTMCQILPKGVVSVLGPSSSPASASTVSHICGEKEIPHIKVGPEETPRLQYLRFASVSLYPSNEDVSLAVS

**KA2(1-100):**

MPAELLLLLIVAFANPSCQVLSSLRMAAILDDQTVCGRGERLALALAREQINGIIEVPAKARVEVDIFELQRDSQYETTDTMCQILPKGVVSVLGPSSSP

**RPL10:**

SGRTQISSSSFEFSSKVSRDTLYEAVREVLHGNQRKRRKFLETVELQISLKNYDPQKDKRFSGTVRLKSTPRPKFSVCVLGDQQHCDEAKAVDIPHMDIEALKKLNKNKKLVKKLAKKYDAFLASESLIKQIPRILGPGLNKAGKFPSLLTHNENMVAKVDEVKSTIKFQMKKVLCLAVAVGHVKMTDDELVYNIHLAVNFLVSLLKKNWQNVRALYIKSTMGKPQRLY

**EE-RR:**

LEIEAAFLEQENTALETEVAELEQEVQRLENIVSQYETRYGPLGSLEIRAAFLRRRNTALRTRVAELRQRVQRLRNIVSQYETRYGPL
