# Supplementary material for: A sensitive soma-localized red fluorescent calcium indicator for in vivo imaging of neuronal populations at single-cell resolution
Source: PLoS Biol. 2025 Apr 29;23(4):e3003048. doi: 10.1371/journal.pbio.3003048 (PMC12040222; doi:10.1371/journal.pbio.3003048)
Supplement: S2 Table — (DOCX) [file pbio.3003048.s018.docx]

**S2 Table.** **List of primers used in this study.**

| **Primer** | **Primer sequence (5’-3’)** |
| --- | --- |
| Fw-LSSmOrange-BglII | gacAGATCTATGGTGAGCAAGGGCGAGGAG |
| RSP-EcoRI-r | CTGGAATTCCTTGGAAGAAATGACACGAGAAAATTCTTCGTAGTTGATTAC |
| CaMSp-M13 | CGTGTCATTTCTTCCAAGGGCGGTTCAGGAGGCAGCGGGTCAggtTCAATGAGGAAACCGTTCCGTG |
| CaMSp-M13-r | CACGGAACGGTTTCCTCATTGAaccTGACCCGCTGCCTCCTGAACCGCCCTTGGAAGAAATGACACG |
| RSPi-EcoRI-r | GATgaattcCTACTTGTACAGCGCGTCCGTG |
| CAG-XCaMP-R-F | gcaaagaattggatccggtaccgccaccatgggttctcatcatcatcatcatcatggtatgg |
| P2A-XCaMP-R-R | TAGCCCCACTTCCCACGTGAACCGGTCGtgggttggactccacgtctcccg |
| CAG-NES-F | tggcaaagaattggatccggtaccgccaccATGCTTCAACTTCCTCCTCTTGAACG |
| P2A-FRCaMPi-R | CCACTTCCCACGTGAACCGGTCGCTTGTACAGCGCGTCCGTGCCAC |
|  |  |
|  |  |
| Syn-NES-F | AGAGCGCAGTCGAGAGGATCCtaagccaccATGCTTCAACTTCCTCCTCTTGAACG |
| FRCaMPi-R | CTTGTACAGCGCGTCCGTGC |
| Linker27-F | GACGCGCTGTACAAGGGCGGATCTGGCGGTAGCGGAGGGACAGGTGGCTCTGGAGGGAGCGGAGGCACTGGCGGATCTGGTGGGAGCGGAGGTACCtcaggccggactcag |
| WPRE- Linker27-R | ATCGATAAGCTTTGTACGGAATTCctaatacagacgctggggcttgcccatg |
| Syn-XCaMP-R-F | CTGAGAGCGCAGTCGAGAGGATCCtaagccaccatgggttctcatcatcatcatcatcatggtatgg |
| Linker27-XCaMPR-R | CCGCTACCGCCAGATCCGCCcttcgctgtcatcatttgtacaaactcttcgtag |
| Syn-NES-jRGECO1a-F | CTGAGAGCGCAGTCGAGAGGATCCtaagccaccatgctgcagaacgagcttgctc |
| Linker27-jRGECO1a-R | CCGCTACCGCCAGATCCGCCcttcgctgtcatcatttgtacaaactcttcgtag |
| Linker27-K-GECO1-R | CCGCTACCGCCAGATCCGCCcttcgctgtcatcatttgtacaaactcttcgtagtttacc |
