# Supplementary material for: A sensitive soma-localized red fluorescent calcium indicator for in vivo imaging of neuronal populations at single-cell resolution
Source: PLoS Biol. 2025 Apr 29;23(4):e3003048. doi: 10.1371/journal.pbio.3003048 (PMC12040222; doi:10.1371/journal.pbio.3003048)
Supplement: S3 Table — (DOCX) [file pbio.3003048.s019.docx]

**S3 Table.** **Summarized In vitro properties of FRCaMPi compared to FRCaMP.**

| **Properties** | | | | **Proteins** | | | | |
| --- | --- | --- | --- | --- | --- | --- | --- | --- |
|  |  |  |  | **FRCaMPi** | | | **FRCaMP^a^** | |
|  |  |  |  | **apo** | **sat** | | **apo** | **sat** |
| **Absorption maximum (nm)** | | | | 447 (573) | 565 | | 446 (575) | 566 |
| **Excitation maximum (nm)** | | | | NF^b^ (576) | 566 | | NF (576) | 564 |
| **Emission maximum (nm)** | | | | NF (600) | 594 | | NF (602) | 592 |
| **Quantum yield ^c^** | | | | <0.004  (0.12±0.01) | 0.231±0.017 | | <0.004  (0.133±0.003) | 0.228±0.008 |
| **e (mM^-1^ cm^-1^) ^d^** | | | | 27.89±0.08 (6.93±0.10) | 50.2±3.3 | | 26.16±0.08 (6.47±0.07) | 53±2 |
| **Brightness vs EGFP (%) ^e^** | | | | 0 (2.5) | 35 | | 0 (2.5) | 36 |
| **DF/F** | **Purified protein** | | **0 mM Mg^2+^** | 16.3±0.3 | | | 16.4±0.7 | |
|  |  |  | **1 mM Mg^2+^** | 16.6±0.8 | | | 15.8±0.5 | |
|  | **HeLa cells** | | | 10±11 | | | 5.6±2.7 | |
| **pKa** | | | | 8.98±0.07 | | 6.48±0.02 | 8.88±0.05 | 6.60±0.04 |
| **K_d_**  **(nM) ^f^** | | **0 mM Mg^2+^** | | 81±2  [n=3.1±0.2] | | | 214±6  [n=2.5±0.2] | |
|  |  | **1 mM Mg^2+^** | | 162±7  [n=2.3±0.2] | | | 441±19  [n=2.7±0.3] | |
| **Maturation half-time (min) ^g^** | | | | 110 | | | ND | |
| **Photobleaching half-time (s) ^h^** | | | | 16.7±9.2 | | | 25.4±7.1* | |

^a^ Data from^24^. ^b^ NF, non-fluorescent. ^c^ Quantum yields (QYs) were determined at pH 7.20. mCherry (QY=0.22 ^76^) and mTagBFP2 (QY=0.64 ^70^) were used as reference standards. ^d^ The extinction coefficient (ε) for the form with an absorption maximum at 565 nm was determined by alkaline denaturation. ^e^ Brightness was calculated as a product of the quantum yield and extinction coefficient and normalized to the brightness of EGFP, which has an extinction coefficient of 56,000 M^−1^·cm^−1^ and a quantum yield of 0.6 ^77^. ^f^ The Hill coefficient is shown in brackets. ^g^ EGFP had a maturation half-time of 14 min. ^h^ Half-time to bleaching up to 50%. One-photon photobleaching was performed under a mercury lamp with drops in oil. Standard deviations are shown.
