# Supplementary material for: A sensitive soma-localized red fluorescent calcium indicator for in vivo imaging of neuronal populations at single-cell resolution
Source: PLoS Biol. 2025 Apr 29;23(4):e3003048. doi: 10.1371/journal.pbio.3003048 (PMC12040222; doi:10.1371/journal.pbio.3003048)
Supplement: S4 Table — (DOCX) [file pbio.3003048.s020.docx]

**S4 Table. Statistics on FRCaMPi characterization in primary hippocampal neurons.**

**Descriptive Statistics on raw data for Peak ΔF/F_0_ comparison (For Fig 2E)**

|  | **K-GECO1** | | | | | | |
| --- | --- | --- | --- | --- | --- | --- | --- |
| **# of Pulses** | **Mean** | **SEM** | **SD** | **N** | **Median** | **Upper limit (Q3)** | **Lower limit (Q1)** |
| 1 | 0.532161423 | 0.034933029 | 0.251905653 | 52 | 0.4899355 | 0.6590115 | 0.380148 |
| 2 | 0.648424333 | 0.03755176 | 0.275947954 | 54 | 0.5981775 | 0.76712025 | 0.46845675 |
| 3 | 0.719743056 | 0.038175157 | 0.28052897 | 54 | 0.671057 | 0.86720325 | 0.5390705 |
| 5 | 0.821271352 | 0.045879061 | 0.337140871 | 54 | 0.753878 | 0.96986225 | 0.606163 |
| 10 | 0.97145237 | 0.055170352 | 0.405417631 | 54 | 0.894426 | 1.136631 | 0.73478575 |
| 20 | 1.092628 | 0.070839073 | 0.515716235 | 53 | 0.970869 | 1.306759 | 0.8192385 |
| 40 | 1.313161981 | 0.087791472 | 0.639131562 | 53 | 1.156186 | 1.6187315 | 0.969852 |
| 80 | 1.677968352 | 0.093669755 | 0.688329313 | 54 | 1.5489965 | 1.9458265 | 1.2402575 |
| 160 | 2.138726755 | 0.092633112 | 0.674379236 | 53 | 2.067695 | 2.420707 | 1.7184945 |

|  | **XCaMP-R** | | | | | | |
| --- | --- | --- | --- | --- | --- | --- | --- |
| **# of Pulses** | **Mean** | **SEM** | **SD** | **N** | **Median** | **Upper limit (Q3)** | **Lower limit (Q1)** |
| 1 | 0.941103125 | 0.083443171 | 0.236012927 | 8 | 0.9788475 | 1.09969475 | 0.78848875 |
| 2 | 0.9852706 | 0.051642253 | 0.163307144 | 10 | 1.0081415 | 1.103374 | 0.816694 |
| 3 | 1.012908769 | 0.062347435 | 0.224796872 | 13 | 1.08987 | 1.132793 | 0.902968 |
| 5 | 1.155260133 | 0.09478605 | 0.367104793 | 15 | 1.051028 | 1.261814 | 0.87872 |
| 10 | 1.12146625 | 0.09982141 | 0.399285641 | 16 | 0.99873 | 1.246739 | 0.8585165 |
| 20 | 1.123915353 | 0.090093963 | 0.371466927 | 17 | 0.985097 | 1.4110415 | 0.8738175 |
| 40 | 1.096731167 | 0.097448813 | 0.33757259 | 12 | 0.9522235 | 1.441464 | 0.88028125 |
| 80 | 1.206415083 | 0.105913035 | 0.366893514 | 12 | 1.29606 | 1.5046325 | 0.894142 |
| 160 | 1.415681444 | 0.24163871 | 0.724916131 | 9 | 1.512597 | 1.9146665 | 0.741307 |

|  | **jRGECO1a** | | | | | | |
| --- | --- | --- | --- | --- | --- | --- | --- |
| **# of Pulses** | **Mean** | **SEM** | **SD** | **N** | **Median** | **Upper limit (Q3)** | **Lower limit (Q1)** |
| 1 | 0.496597658 | 0.055024975 | 0.339196724 | 38 | 0.345808 | 0.65764175 | 0.24424075 |
| 2 | 0.77603725 | 0.098181343 | 0.589088057 | 36 | 0.5211835 | 1.0100615 | 0.364779 |
| 3 | 0.985442946 | 0.12508574 | 0.76086685 | 37 | 0.733023 | 1.4066665 | 0.4511145 |
| 5 | 1.304196711 | 0.168353567 | 1.037801088 | 38 | 0.877511 | 1.7341845 | 0.497647 |
| 10 | 1.440519938 | 0.144539073 | 1.156312581 | 64 | 1.048791 | 1.707884 | 0.74840175 |
| 20 | 1.945200395 | 0.250684303 | 1.545321826 | 38 | 1.418707 | 2.5260885 | 0.75112425 |
| 40 | 1.93121446 | 0.18019563 | 1.43025847 | 63 | 1.494065 | 2.481812 | 0.862112 |
| 80 | 2.564210381 | 0.253369565 | 2.011058578 | 63 | 2.146777 | 2.8809 | 1.165745 |
| 160 | 3.287889339 | 0.305174162 | 2.402943755 | 62 | 2.5266315 | 3.95946325 | 1.783843 |

|  | **FRCaMPi** | | | | | | |
| --- | --- | --- | --- | --- | --- | --- | --- |
| **# of Pulses** | **Mean** | **SEM** | **SD** | **N** | **Median** | **Upper limit (Q3)** | **Lower limit (Q1)** |
| 1 | 0.820256664 | 0.103535606 | 0.732107293 | 50 | 0.581232 | 0.96064125 | 0.38380025 |
| 2 | 1.373174331 | 0.170306998 | 1.228101229 | 52 | 1.0567705 | 1.749476 | 0.59730575 |
| 3 | 1.619348649 | 0.204853167 | 1.433972166 | 49 | 1.245736 | 1.9202705 | 0.714798 |
| 5 | 2.276478025 | 0.309759571 | 2.212125804 | 51 | 1.513434 | 2.58595 | 0.98573 |
| 10 | 2.649220212 | 0.337174745 | 2.40790931 | 51 | 1.900541 | 3.063639 | 1.294712 |
| 20 | 2.954032578 | 0.278641064 | 2.047585287 | 54 | 2.573126 | 3.5016195 | 1.676454 |
| 40 | 3.365055104 | 0.297908854 | 2.106533706 | 50 | 2.751984 | 4.1425625 | 2.09519425 |
| 80 | 3.90234227 | 0.267427255 | 1.965180957 | 54 | 3.5409745 | 5.0088125 | 2.579926 |
| 160 | 4.841294704 | 0.399279297 | 2.879243959 | 52 | 3.9711768 | 7.42190325 | 2.94680675 |

**Two-way ANOVA analysis for Peak ΔF/F_0_ comparison (Fig 2E)**

| **Mixed-effects model (REML)** | **Matching: Stacked** |  |  |  |  |
| --- | --- | --- | --- | --- | --- |
| Assume sphericity? | No |  |  |  |  |
| Alpha | 0.05 |  |  |  |  |
|  |  |  |  |  |  |
| **Fixed effects (type III)** | **P value** | **P value summary** | **Statistically significant (P < 0.05)?** | **F (DFn, DFd)** | **Geisser-Greenhouse's epsilon** |
| # of Pulses | <0.000000000000001 | **** | Yes | F (2.553, 405.0) = 70.03 | 0.3192 |
| Red GECIs | 0.000000000003196 | **** | Yes | F (3, 190) = 21.89 |  |
| # of Pulses x Red GECIs | <0.000000000000001 | **** | Yes | F (24, 1269) = 11.61 |  |
|  |  |  |  |  |  |
| **Random effects** | **SD** | **Variance** |  |  |  |
| Subject | 1.166 | 1.360 |  |  |  |
| Residual | 0.7840 | 0.6147 |  |  |  |
|  |  |  |  |  |  |
| **Was the matching effective?** |  |  |  |  |  |
| Chi-square, df | 1176, 1 |  |  |  |  |
| P value | <0.000000000000001 |  |  |  |  |
| P value summary | **** |  |  |  |  |
| Is there significant matching (P < 0.05)? | Yes |  |  |  |  |
|  |  |  |  |  |  |
| **Data summary** |  |  |  |  |  |
| Number of columns (# of Pulses) | 4 |  |  |  |  |
| Number of rows (Red GECI) | 9 |  |  |  |  |
| Number of subjects (Subject) | 194 |  |  |  |  |
| Number of missing values | 251 |  |  |  |  |

**Dunnett post-hoc analysis for Peak ΔF/F_0_ comparison (Fig 2E)**

|  | **Mean Diff.** | **95.00% CI of diff.** | **SE of diff.** | **q** | **DF** | **Adjusted P Value** | **Summary** |
| --- | --- | --- | --- | --- | --- | --- | --- |
| **# of Pulses:** |  |  |  |  |  |  |  |
| **1** |  |  |  |  |  |  |  |
| FRCaMPi vs. K-GECO1 | 0.2881 | 0.02214 to 0.5540 | 0.1093 | 2.637 | 60.04 | 0.030132088925053 | * |
| FRCaMPi vs. XCaMP-R | -0.1208 | -0.4513 to 0.2096 | 0.1330 | 0.9088 | 33.73 | 0.718896212433823 | ns |
| FRCaMPi vs. jRGECO1a | 0.3237 | 0.03962 to 0.6077 | 0.1172 | 2.760 | 72.89 | 0.020865914283679 | * |
|  |  |  |  |  |  |  |  |
| **2** |  |  |  |  |  |  |  |
| FRCaMPi vs. K-GECO1 | 0.7247 | 0.2995 to 1.150 | 0.1744 | 4.156 | 55.95 | 0.000332730438936 | *** |
| FRCaMPi vs. XCaMP-R | 0.3879 | -0.04561 to 0.8214 | 0.1780 | 2.180 | 58.03 | 0.090620029582555 | ns |
| FRCaMPi vs. jRGECO1a | 0.5971 | 0.1216 to 1.073 | 0.1966 | 3.038 | 77.98 | 0.009405582258819 | ** |
|  |  |  |  |  |  |  |  |
| **3** |  |  |  |  |  |  |  |
| FRCaMPi vs. K-GECO1 | 0.8996 | 0.3910 to 1.408 | 0.2084 | 4.317 | 51.34 | 0.000214140746521 | *** |
| FRCaMPi vs. XCaMP-R | 0.6064 | 0.08500 to 1.128 | 0.2141 | 2.832 | 55.40 | 0.018264047387059 | * |
| FRCaMPi vs. jRGECO1a | 0.6339 | 0.05399 to 1.214 | 0.2400 | 2.641 | 76.32 | 0.028219730317632 | * |
|  |  |  |  |  |  |  |  |
| **5** |  |  |  |  |  |  |  |
| FRCaMPi vs. K-GECO1 | 1.455 | 0.6914 to 2.219 | 0.3131 | 4.647 | 52.19 | 0.000069023968620 | **** |
| FRCaMPi vs. XCaMP-R | 1.121 | 0.3335 to 1.909 | 0.3239 | 3.461 | 57.99 | 0.002963855316385 | ** |
| FRCaMPi vs. jRGECO1a | 0.9723 | 0.1203 to 1.824 | 0.3526 | 2.758 | 75.05 | 0.020714018114619 | * |
|  |  |  |  |  |  |  |  |
| **10** |  |  |  |  |  |  |  |
| FRCaMPi vs. K-GECO1 | 1.678 | 0.8471 to 2.508 | 0.3417 | 4.911 | 52.68 | 0.000027262528581 | **** |
| FRCaMPi vs. XCaMP-R | 1.528 | 0.6750 to 2.380 | 0.3516 | 4.345 | 57.67 | 0.000168914568671 | *** |
| FRCaMPi vs. jRGECO1a | 1.209 | 0.3228 to 2.095 | 0.3668 | 3.295 | 68.24 | 0.004501630361904 | ** |
|  |  |  |  |  |  |  |  |
| **20** |  |  |  |  |  |  |  |
| FRCaMPi vs. K-GECO1 | 1.861 | 1.163 to 2.560 | 0.2875 | 6.474 | 59.82 | 0.000000059309122 | **** |
| FRCaMPi vs. XCaMP-R | 1.830 | 1.119 to 2.541 | 0.2928 | 6.249 | 62.40 | 0.000000123059526 | **** |
| FRCaMPi vs. jRGECO1a | 1.009 | 0.1059 to 1.912 | 0.3748 | 2.692 | 89.51 | 0.024052215381437 | * |
|  |  |  |  |  |  |  |  |
| **40** |  |  |  |  |  |  |  |
| FRCaMPi vs. K-GECO1 | 2.052 | 1.298 to 2.806 | 0.3106 | 6.607 | 57.47 | 0.000000041517259 | **** |
| FRCaMPi vs. XCaMP-R | 2.268 | 1.507 to 3.029 | 0.3134 | 7.237 | 57.13 | 0.000000003794144 | **** |
| FRCaMPi vs. jRGECO1a | 1.434 | 0.5956 to 2.272 | 0.3482 | 4.118 | 82.67 | 0.000266135506630 | *** |
|  |  |  |  |  |  |  |  |
| **80** |  |  |  |  |  |  |  |
| FRCaMPi vs. K-GECO1 | 2.224 | 1.538 to 2.911 | 0.2834 | 7.850 | 65.81 | 0.000000000149846 | **** |
| FRCaMPi vs. XCaMP-R | 2.696 | 1.999 to 3.393 | 0.2876 | 9.373 | 63.41 | 0.000000000000408 | **** |
| FRCaMPi vs. jRGECO1a | 1.338 | 0.4549 to 2.221 | 0.3684 | 3.632 | 113.0 | 0.001246362709749 | ** |
|  |  |  |  |  |  |  |  |
| **160** |  |  |  |  |  |  |  |
| FRCaMPi vs. K-GECO1 | 2.703 | 1.705 to 3.700 | 0.4099 | 6.593 | 56.48 | 0.000000046885442 | **** |
| FRCaMPi vs. XCaMP-R | 3.426 | 2.287 to 4.564 | 0.4667 | 7.340 | 51.32 | 0.000000004576725 | **** |
| FRCaMPi vs. jRGECO1a | 1.553 | 0.3455 to 2.761 | 0.5025 | 3.091 | 99.58 | 0.007474374263449 | ** |

**Descriptive Statistics on raw data for Peak SNR comparison (Fig 2F)**

|  | **K-GECO1** | | | | | | |
| --- | --- | --- | --- | --- | --- | --- | --- |
| **# of Pulses** | **Mean** | **SEM** | **SD** | **N** | **Median** | **Upper limit (Q3)** | **Lower limit (Q1)** |
| 1 | 31.117954 | 2.0861304 | 14.75117 | 50 | 28.290205 | 35.99621 | 21.167683 |
| 2 | 39.630607 | 2.6798331 | 19.324551 | 52 | 33.94791 | 48.57845 | 28.483558 |
| 3 | 48.236028 | 3.5267257 | 23.919418 | 46 | 42.857745 | 59.294168 | 33.652705 |
| 5 | 50.833538 | 3.5183614 | 25.126126 | 51 | 44.79283 | 55.20598 | 35.61729 |
| 10 | 66.979848 | 5.0818072 | 36.645433 | 52 | 57.18124 | 77.04459 | 42.231148 |
| 20 | 71.453171 | 5.3380535 | 36.595851 | 47 | 64.29375 | 79.07261 | 49.5303 |
| 40 | 84.650218 | 5.3135247 | 36.813179 | 48 | 81.729 | 101.1691 | 58.871908 |
| 80 | 118.28464 | 7.6280057 | 55.006331 | 52 | 105.6638 | 140.16778 | 83.206345 |
| 160 | 157.47437 | 12.949074 | 83.919592 | 42 | 135.14925 | 172.0135 | 102.783 |

|  | **XCaMP-R** | | | | | | |
| --- | --- | --- | --- | --- | --- | --- | --- |
| **# of Pulses** | **Mean** | **SEM** | **SD** | **N** | **Median** | **Upper limit (Q3)** | **Lower limit (Q1)** |
| 1 | 33.969596 | 4.4730457 | 12.651684 | 8 | 30.600685 | 44.676363 | 27.049708 |
| 2 | 49.523974 | 4.8243167 | 15.255829 | 10 | 46.53035 | 63.428365 | 37.748125 |
| 3 | 38.547058 | 4.6419232 | 16.736692 | 13 | 33.69093 | 49.35978 | 24.693545 |
| 5 | 44.708994 | 4.6584464 | 18.042085 | 15 | 37.48438 | 52.51416 | 33.27577 |
| 10 | 41.429466 | 4.0172095 | 16.068838 | 16 | 36.99338 | 51.099583 | 30.026278 |
| 20 | 38.092454 | 2.7717558 | 11.087023 | 16 | 38.573975 | 47.562665 | 26.625393 |
| 40 | 34.682541 | 3.9678377 | 13.744993 | 12 | 33.029465 | 36.941575 | 26.2589 |
| 80 | 32.034206 | 2.6389068 | 9.1414414 | 12 | 30.1974 | 38.394748 | 24.173163 |
| 160 | 49.287134 | 9.4473202 | 29.87505 | 10 | 47.00383 | 73.398484 | 20.09493 |

|  | **jRGECO1a** | | | | | | |
| --- | --- | --- | --- | --- | --- | --- | --- |
| **# of Pulses** | **Mean** | **SEM** | **SD** | **N** | **Median** | **Upper limit (Q3)** | **Lower limit (Q1)** |
| 1 | 42.831012 | 4.2813719 | 26.737159 | 39 | 49.35821 | 61.38771 | 13.13559 |
| 2 | 81.243969 | 4.8320554 | 29.78679 | 38 | 77.705165 | 101.4016 | 55.576135 |
| 3 | 93.625747 | 6.8562552 | 41.137531 | 36 | 92.89635 | 117.8282 | 62.485158 |
| 5 | 116.81406 | 9.3352588 | 57.5464 | 38 | 106.2998 | 151.61758 | 78.952015 |
| 10 | 139.27596 | 8.6646488 | 69.31719 | 64 | 133.1026 | 165.09515 | 89.98892 |
| 20 | 182.85332 | 14.094086 | 88.017536 | 39 | 184.2403 | 230.8262 | 103.5264 |
| 40 | 190.10118 | 12.626949 | 101.80172 | 65 | 169.0824 | 243.13455 | 109.5487 |
| 80 | 241.54697 | 14.734039 | 107.26542 | 53 | 234.053 | 329.9811 | 164.587 |
| 160 | 330.46 | 20.068178 | 150.17649 | 56 | 291.8251 | 396.89115 | 228.94215 |

|  | **FRCaMPi** | | | | | | |
| --- | --- | --- | --- | --- | --- | --- | --- |
| **# of Pulses** | **Mean** | **SEM** | **SD** | **N** | **Median** | **Upper limit (Q3)** | **Lower limit (Q1)** |
| 1 | 50.192914 | 5.4392152 | 39.222739 | 52 | 37.213025 | 66.698143 | 23.055373 |
| 2 | 62.288658 | 4.8605861 | 34.024103 | 49 | 60.69378 | 84.77657 | 36.57484 |
| 3 | 71.291895 | 5.7460386 | 39.809723 | 48 | 63.83349 | 100.18879 | 40.67588 |
| 5 | 106.36994 | 12.248687 | 84.86139 | 48 | 80.817865 | 136.90178 | 58.506195 |
| 10 | 108.64897 | 11.199783 | 72.582893 | 42 | 93.83085 | 123.6864 | 64.23942 |
| 20 | 121.69943 | 8.5769932 | 62.441453 | 53 | 109.4512 | 150.7675 | 76.005705 |
| 40 | 148.86599 | 9.92374 | 72.924298 | 54 | 124.4409 | 186.30945 | 97.077185 |
| 80 | 183.38326 | 12.622914 | 94.461237 | 56 | 153.30205 | 236.41728 | 118.64438 |
| 160 | 254.31988 | 23.099928 | 130.67293 | 32 | 236.88765 | 359.2187 | 145.54015 |

**Two-way ANOVA analysis for Peak SNR comparison (Fig 2F)**

| **Mixed-effects model (REML)** | **Matching: Stacked** |  |  |  |  |
| --- | --- | --- | --- | --- | --- |
| Assume sphericity? | No |  |  |  |  |
| Alpha | 0.05 |  |  |  |  |
|  |  |  |  |  |  |
| **Fixed effects (type III)** | **P value** | **P value summary** | **Statistically significant (P < 0.05)?** | **F (DFn, DFd)** | **Geisser-Greenhouse's epsilon** |
| # of Pulses | <0.000000000000001 | **** | Yes | F (2.696, 400.0) = 100.4 | 0.3370 |
| Red GECIs | 0.000000000000006 | **** | Yes | F (3, 191) = 27.71 |  |
| # of Pulses x Red GECIs | <0.000000000000001 | **** | Yes | F (24, 1187) = 16.97 |  |
|  |  |  |  |  |  |
| **Random effects** | **SD** | **Variance** |  |  |  |
| Subject | 51.22 | 2623 |  |  |  |
| Residual | 46.34 | 2148 |  |  |  |
|  |  |  |  |  |  |
| **Was the matching effective?** |  |  |  |  |  |
| Chi-square, df | 626.4, 1 |  |  |  |  |
| P value | <0.000000000000001 |  |  |  |  |
| P value summary | **** |  |  |  |  |
| Is there significant matching (P < 0.05)? | Yes |  |  |  |  |
|  |  |  |  |  |  |
| **Data summary** |  |  |  |  |  |
| Number of columns (# of Pulses) | 4 |  |  |  |  |
| Number of rows (Red GECI) | 9 |  |  |  |  |
| Number of subjects (Subject) | 195 |  |  |  |  |
| Number of missing values | 341 |  |  |  |  |

**Dunnett post-hoc analysis for Peak SNR comparison (Fig 2F)**

|  | **Mean Diff.** | **95.00% CI of diff.** | **SE of diff.** | **q** | **DF** | **Adjusted P Value** | **Summary** |
| --- | --- | --- | --- | --- | --- | --- | --- |
| **# of Pulses:** |  |  |  |  |  |  |  |
| **1** |  |  |  |  |  |  |  |
| FRCaMPi vs. K-GECO1 | 19.07 | 4.922 to 33.23 | 5.826 | 3.274 | 65.63 | 0.004949039442883 | ** |
| FRCaMPi vs. XCaMP-R | 16.22 | -1.304 to 33.75 | 7.042 | 2.304 | 33.08 | 0.075500407376634 | ns |
| FRCaMPi vs. jRGECO1a | 7.362 | -9.350 to 24.07 | 6.922 | 1.064 | 88.29 | 0.613570610145662 | ns |
|  |  |  |  |  |  |  |  |
| **2** |  |  |  |  |  |  |  |
| FRCaMPi vs. K-GECO1 | 22.66 | 9.232 to 36.08 | 5.550 | 4.082 | 75.09 | 0.000327288448623 | *** |
| FRCaMPi vs. XCaMP-R | 12.76 | -4.324 to 29.85 | 6.848 | 1.864 | 30.63 | 0.183874453570144 | ns |
| FRCaMPi vs. jRGECO1a | -18.96 | -35.50 to -2.412 | 6.854 | 2.766 | 83.70 | 0.019944020041827 | * |
|  |  |  |  |  |  |  |  |
| **3** |  |  |  |  |  |  |  |
| FRCaMPi vs. K-GECO1 | 23.06 | 6.764 to 39.35 | 6.742 | 3.420 | 77.58 | 0.002936063393826 | ** |
| FRCaMPi vs. XCaMP-R | 32.74 | 14.67 to 50.82 | 7.387 | 4.433 | 48.11 | 0.000159413788670 | *** |
| FRCaMPi vs. jRGECO1a | -22.33 | -43.97 to -0.6960 | 8.946 | 2.497 | 74.18 | 0.041205444770320 | * |
|  |  |  |  |  |  |  |  |
| **5** |  |  |  |  |  |  |  |
| FRCaMPi vs. K-GECO1 | 55.54 | 24.51 to 86.56 | 12.74 | 4.358 | 54.73 | 0.000172691573149 | *** |
| FRCaMPi vs. XCaMP-R | 61.66 | 29.81 to 93.52 | 13.10 | 4.705 | 57.54 | 0.000048638697953 | **** |
| FRCaMPi vs. jRGECO1a | -10.44 | -47.57 to 26.68 | 15.40 | 0.6782 | 82.22 | 0.852077065272053 | ns |
|  |  |  |  |  |  |  |  |
| **10** |  |  |  |  |  |  |  |
| FRCaMPi vs. K-GECO1 | 41.67 | 11.93 to 71.41 | 12.30 | 3.388 | 57.66 | 0.003632465876486 | ** |
| FRCaMPi vs. XCaMP-R | 67.22 | 38.33 to 96.11 | 11.90 | 5.649 | 49.97 | 0.000002271473363 | **** |
| FRCaMPi vs. jRGECO1a | -30.63 | -64.57 to 3.314 | 14.16 | 2.163 | 84.96 | 0.086555594848898 | ns |
|  |  |  |  |  |  |  |  |
| **20** |  |  |  |  |  |  |  |
| FRCaMPi vs. K-GECO1 | 50.25 | 25.88 to 74.61 | 10.10 | 4.974 | 85.57 | 0.000010016209118 | **** |
| FRCaMPi vs. XCaMP-R | 83.61 | 61.70 to 105.5 | 9.014 | 9.276 | 61.12 | 0.000000000000858 | **** |
| FRCaMPi vs. jRGECO1a | -61.15 | -101.2 to -21.12 | 16.50 | 3.707 | 64.86 | 0.001287325492789 | ** |
|  |  |  |  |  |  |  |  |
| **40** |  |  |  |  |  |  |  |
| FRCaMPi vs. K-GECO1 | 64.22 | 37.06 to 91.37 | 11.26 | 5.705 | 80.30 | 0.000000561880578 | **** |
| FRCaMPi vs. XCaMP-R | 114.2 | 88.27 to 140.1 | 10.69 | 10.68 | 63.48 | <0.000000000000001 | **** |
| FRCaMPi vs. jRGECO1a | -41.24 | -79.75 to -2.721 | 16.06 | 2.568 | 114.7 | 0.032317653434775 | * |
|  |  |  |  |  |  |  |  |
| **80** |  |  |  |  |  |  |  |
| FRCaMPi vs. K-GECO1 | 65.10 | 29.56 to 100.6 | 14.75 | 4.414 | 89.62 | 0.000084167774503 | **** |
| FRCaMPi vs. XCaMP-R | 151.3 | 120.0 to 182.7 | 12.90 | 11.74 | 59.35 | 0.000000000000012 | **** |
| FRCaMPi vs. jRGECO1a | -58.16 | -104.8 to -11.52 | 19.40 | 2.998 | 103.6 | 0.009835692627359 | ** |
|  |  |  |  |  |  |  |  |
| **160** |  |  |  |  |  |  |  |
| FRCaMPi vs. K-GECO1 | 96.85 | 32.55 to 161.1 | 26.48 | 3.657 | 49.82 | 0.001763124603335 | ** |
| FRCaMPi vs. XCaMP-R | 205.0 | 143.9 to 266.2 | 24.96 | 8.215 | 38.53 | 0.000000001600993 | **** |
| FRCaMPi vs. jRGECO1a | -76.14 | -149.7 to -2.567 | 30.60 | 2.488 | 72.25 | 0.040683563821684 | * |
