# Supplementary material for: A sensitive soma-localized red fluorescent calcium indicator for in vivo imaging of neuronal populations at single-cell resolution
Source: PLoS Biol. 2025 Apr 29;23(4):e3003048. doi: 10.1371/journal.pbio.3003048 (PMC12040222; doi:10.1371/journal.pbio.3003048)
Supplement: S5 Table — (DOCX) [file pbio.3003048.s021.docx]

**S5 Table. Statistics on SomaFRCaMPi characterization in primary hippocampal neurons**

**Multiple unpaired t tests for characterizing fluorescence intensity along the neurite (Fig 3D-G)**

**K-GECO1 vs. SomaK-GECO1 (Fig 3D)**

**Test details:**

| **Test name** | Unpaired t test |
| --- | --- |
| **Variance assumption** | Individual variance for each row |
| **Multiple comparisons** | Set P value threshold |
| **Method** | No correction for multiple comparisons |
| **Alpha** | 0.05 |
|  |  |
| **Number of tests performed** | 10 |
| **Number of rows omitted** | 1 |

**t-test results:**

| **Distance to Soma (μm)** | **Below threshold?** | **P value** | **Mean Difference** | **SE of difference** | **t ratio** | **df** |
| --- | --- | --- | --- | --- | --- | --- |
| Soma |  |  | 0.000 | 0.000 |  |  |
| 10 | No | 0.070788 | 0.1871 | 0.09356 | 2.000 | 11.00 |
| 20 | Yes | 0.013204 | 0.1913 | 0.06485 | 2.950 | 11.00 |
| 30 | Yes | 0.000103 | 0.2687 | 0.04556 | 5.898 | 11.00 |
| 40 | Yes | 0.002321 | 0.2799 | 0.07109 | 3.938 | 11.00 |
| 50 | Yes | 0.000111 | 0.2306 | 0.03941 | 5.851 | 11.00 |
| 60 | Yes | 0.000057 | 0.2898 | 0.04590 | 6.313 | 11.00 |
| 70 | Yes | 0.000745 | 0.1978 | 0.04285 | 4.616 | 11.00 |
| 80 | Yes | 0.001251 | 0.2455 | 0.05706 | 4.302 | 11.00 |
| 90 | Yes | 0.000055 | 0.2190 | 0.03454 | 6.342 | 11.00 |
| 100 | Yes | 0.000003 | 0.2040 | 0.02387 | 8.549 | 11.00 |

**XCaMP-R vs. SomaXCaMP-R (Fig 3E)**

**Test details:**

| **Test name** | Unpaired t test |
| --- | --- |
| **Variance assumption** | Individual variance for each row |
| **Multiple comparisons** | Set P value threshold |
| **Method** | No correction for multiple comparisons |
| **Alpha** | 0.05 |
|  |  |
| **Number of tests performed** | 10 |
| **Number of rows omitted** | 1 |

**t-test results:**

| **Distance to Soma (μm)** | **Below threshold?** | **P value** | **Mean Difference** | **SE of difference** | **t ratio** | **df** |
| --- | --- | --- | --- | --- | --- | --- |
| 0 |  |  | 0.000 | 0.000 |  |  |
| 10 | No | 0.147841 | 0.2223 | 0.1417 | 1.568 | 10.00 |
| 20 | Yes | 0.002641 | 0.2862 | 0.07209 | 3.971 | 10.00 |
| 30 | Yes | 0.002499 | 0.2304 | 0.05753 | 4.005 | 10.00 |
| 40 | Yes | 0.001027 | 0.2536 | 0.05550 | 4.570 | 10.00 |
| 50 | Yes | 0.002043 | 0.2594 | 0.06281 | 4.130 | 10.00 |
| 60 | Yes | 0.013538 | 0.1750 | 0.05848 | 2.992 | 10.00 |
| 70 | Yes | 0.004318 | 0.1794 | 0.04889 | 3.670 | 10.00 |
| 80 | Yes | 0.003252 | 0.2661 | 0.06926 | 3.843 | 10.00 |
| 90 | Yes | 0.000030 | 0.2648 | 0.03694 | 7.167 | 10.00 |
| 100 | Yes | 0.000011 | 0.2606 | 0.03247 | 8.024 | 10.00 |

**jRGECO1a vs. SomajRGECO1a (Fig 3F)**

**Test details:**

| **Test name** | Unpaired t test |
| --- | --- |
| **Variance assumption** | Individual variance for each row |
| **Multiple comparisons** | Set P value threshold |
| **Method** | No correction for multiple comparisons |
| **Alpha** | 0.05 |
|  |  |
| **Number of tests performed** | 10 |
| **Number of rows omitted** | 1 |

**t-test results:**

| **Distance to Soma (μm)** | **Below threshold?** | **P value** | **Mean Difference** | **SE of difference** | **t ratio** | **df** |
| --- | --- | --- | --- | --- | --- | --- |
| 0 |  |  | 0.000 | 0.000 |  |  |
| 10 | Yes | 0.037063 | 0.2854 | 0.1187 | 2.404 | 10.00 |
| 20 | No | 0.128490 | 0.1783 | 0.1076 | 1.657 | 10.00 |
| 30 | Yes | 0.010678 | 0.2444 | 0.07807 | 3.131 | 10.00 |
| 40 | Yes | 0.005080 | 0.2415 | 0.06762 | 3.572 | 10.00 |
| 50 | Yes | 0.004935 | 0.1865 | 0.05196 | 3.589 | 10.00 |
| 60 | Yes | 0.010058 | 0.1248 | 0.03941 | 3.166 | 10.00 |
| 70 | Yes | 0.001662 | 0.1553 | 0.03644 | 4.260 | 10.00 |
| 80 | Yes | 0.027554 | 0.1772 | 0.06875 | 2.577 | 10.00 |
| 90 | Yes | 0.001896 | 0.2138 | 0.05118 | 4.177 | 10.00 |
| 100 | Yes | 0.001305 | 0.1770 | 0.04008 | 4.415 | 10.00 |

**FRCaMPi vs. SomaFRCaMPi (Fig 3G)**

**Test details:**

| **Test name** | Unpaired t test |
| --- | --- |
| **Variance assumption** | Individual variance for each row |
| **Multiple comparisons** | Set P value threshold |
| **Method** | No correction for multiple comparisons |
| **Desired FDR (Q)** | 0.05 |
|  |  |
| **Number of tests performed** | 10 |
| **Number of rows omitted** | 1 |

**t-test results:**

| **Distance to Soma (μm)** | **Discovery?** | **P value** | **Mean Difference** | **SE of difference** | **t ratio** | **df** |
| --- | --- | --- | --- | --- | --- | --- |
| 0 |  |  | 0.000 | 0.000 |  |  |
| 10 | No | 0.095867 | 0.1589 | 0.08725 | 1.821 | 11.00 |
| 20 | Yes | 0.016546 | 0.2969 | 0.1051 | 2.824 | 11.00 |
| 30 | Yes | 0.008430 | 0.2834 | 0.08852 | 3.202 | 11.00 |
| 40 | Yes | 0.016348 | 0.2998 | 0.1059 | 2.831 | 11.00 |
| 50 | Yes | 0.008583 | 0.3289 | 0.1031 | 3.192 | 11.00 |
| 60 | Yes | 0.030284 | 0.3652 | 0.1469 | 2.485 | 11.00 |
| 70 | Yes | 0.010901 | 0.2844 | 0.09302 | 3.057 | 11.00 |
| 80 | Yes | 0.030639 | 0.2377 | 0.09590 | 2.479 | 11.00 |
| 90 | Yes | 0.019265 | 0.2654 | 0.09689 | 2.739 | 11.00 |
| 100 | Yes | 0.045702 | 0.2086 | 0.09143 | 2.281 | 10.00 |

**Two-way ANOVA analysis for Peak ΔF/F_0_ comparison (Fig 3l)**

| **Mixed-effects model (REML)** | **Matching: Stacked** |  |  |  |  |
| --- | --- | --- | --- | --- | --- |
| Assume sphericity? | No |  |  |  |  |
| Alpha | 0.05 |  |  |  |  |
|  |  |  |  |  |  |
| **Fixed effects (type III)** | **P value** | **P value summary** | **Statistically significant (P < 0.05)?** | **F (DFn, DFd)** | **Geisser-Greenhouse's epsilon** |
| # of Pulses | <0.000000000000001 | **** | Yes | F (2.180, 671.8) = 115.2 | 0.2725 |
| Red GECI | <0.000000000000001 | **** | Yes | F (3, 351) = 85.30 |  |
| # of Pulses x Red GECI | <0.000000000000001 | **** | Yes | F (24, 2465) = 37.60 |  |
|  |  |  |  |  |  |
| **Random effects** | **SD** | **Variance** |  |  |  |
| Subject | 1.425 | 2.030 |  |  |  |
| Residual | 0.8020 | 0.6432 |  |  |  |
|  |  |  |  |  |  |
| **Was the matching effective?** |  |  |  |  |  |
| Chi-square, df | 2661, 1 |  |  |  |  |
| P value | <0.000000000000001 |  |  |  |  |
| P value summary | **** |  |  |  |  |
| Is there significant matching (P < 0.05)? | Yes |  |  |  |  |
|  |  |  |  |  |  |
| **Data summary** |  |  |  |  |  |
| Number of columns (# of Pulses) | 4 |  |  |  |  |
| Number of rows (Red GECI) | 9 |  |  |  |  |
| Number of subjects (Subject) | 355 |  |  |  |  |
| Number of missing values | 343 |  |  |  |  |

**Dunnett post-hoc analysis for Peak ΔF/F_0_ comparison (Fig 3l)**

|  | **Mean Diff.** | **95.00% CI of diff.** | **SE of diff.** | **q** | **DF** | **Adjusted P Value** | **Summary** |
| --- | --- | --- | --- | --- | --- | --- | --- |
| **# of Pulses:** |  |  |  |  |  |  |  |
| **1** |  |  |  |  |  |  |  |
| SomaFRCaMPi vs. SomaK-GECO1 | 1.511 | 1.327 to 1.695 | 0.07650 | 19.75 | 126.2 | 0.000000000000020 | **** |
| SomaFRCaMPi vs. SomaXCaMP-R | 1.156 | 0.9199 to 1.391 | 0.09830 | 11.76 | 149.2 | <0.000000000000001 | **** |
| SomaFRCaMPi vs. SomajRGECO1a | 0.6592 | 0.3685 to 0.9499 | 0.1211 | 5.445 | 134.2 | 0.000000717952742 | **** |
|  |  |  |  |  |  |  |  |
| **2** |  |  |  |  |  |  |  |
| SomaFRCaMPi vs. SomaK-GECO1 | 1.857 | 1.599 to 2.116 | 0.1075 | 17.28 | 120.8 | <0.000000000000001 | **** |
| SomaFRCaMPi vs. SomaXCaMP-R | 1.372 | 1.070 to 1.674 | 0.1261 | 10.88 | 166.2 | <0.000000000000001 | **** |
| SomaFRCaMPi vs. SomajRGECO1a | 0.7218 | 0.09910 to 1.345 | 0.2557 | 2.823 | 61.16 | 0.018468468108122 | * |
|  |  |  |  |  |  |  |  |
| **3** |  |  |  |  |  |  |  |
| SomaFRCaMPi vs. SomaK-GECO1 | 2.211 | 1.927 to 2.496 | 0.1184 | 18.68 | 128.4 | <0.000000000000001 | **** |
| SomaFRCaMPi vs. SomaXCaMP-R | 1.586 | 1.259 to 1.913 | 0.1367 | 11.61 | 180.9 | <0.000000000000001 | **** |
| SomaFRCaMPi vs. SomajRGECO1a | 0.8303 | 0.2668 to 1.394 | 0.2335 | 3.556 | 93.18 | 0.001758541477725 | ** |
|  |  |  |  |  |  |  |  |
| **5** |  |  |  |  |  |  |  |
| SomaFRCaMPi vs. SomaK-GECO1 | 2.792 | 2.431 to 3.153 | 0.1505 | 18.55 | 135.5 | 0.000000000000005 | **** |
| SomaFRCaMPi vs. SomaXCaMP-R | 2.081 | 1.692 to 2.470 | 0.1626 | 12.80 | 170.5 | <0.000000000000001 | **** |
| SomaFRCaMPi vs. SomajRGECO1a | 1.259 | 0.7635 to 1.754 | 0.2074 | 6.069 | 207.5 | 0.000000018050878 | **** |
|  |  |  |  |  |  |  |  |
| **10** |  |  |  |  |  |  |  |
| SomaFRCaMPi vs. SomaK-GECO1 | 3.304 | 2.894 to 3.714 | 0.1709 | 19.33 | 130.5 | <0.000000000000001 | **** |
| SomaFRCaMPi vs. SomaXCaMP-R | 2.428 | 1.989 to 2.867 | 0.1835 | 13.23 | 162.3 | <0.000000000000001 | **** |
| SomaFRCaMPi vs. SomajRGECO1a | 1.650 | 1.088 to 2.212 | 0.2356 | 7.003 | 209.1 | 0.000000000101194 | **** |
|  |  |  |  |  |  |  |  |
| **20** |  |  |  |  |  |  |  |
| SomaFRCaMPi vs. SomaK-GECO1 | 3.588 | 3.139 to 4.036 | 0.1870 | 19.19 | 132.9 | <0.000000000000001 | **** |
| SomaFRCaMPi vs. SomaXCaMP-R | 2.675 | 2.198 to 3.151 | 0.1991 | 13.43 | 163.6 | 0.000000000000019 | **** |
| SomaFRCaMPi vs. SomajRGECO1a | 1.881 | 1.293 to 2.468 | 0.2461 | 7.644 | 220.7 | 0.000000000002063 | **** |
|  |  |  |  |  |  |  |  |
| **40** |  |  |  |  |  |  |  |
| SomaFRCaMPi vs. SomaK-GECO1 | 4.052 | 3.483 to 4.622 | 0.2375 | 17.07 | 131.1 | <0.000000000000001 | **** |
| SomaFRCaMPi vs. SomaXCaMP-R | 3.071 | 2.473 to 3.669 | 0.2498 | 12.29 | 156.5 | 0.000000000000004 | **** |
| SomaFRCaMPi vs. SomajRGECO1a | 2.344 | 1.665 to 3.024 | 0.2846 | 8.236 | 212.9 | 0.000000000000045 | **** |
|  |  |  |  |  |  |  |  |
| **80** |  |  |  |  |  |  |  |
| SomaFRCaMPi vs. SomaK-GECO1 | 4.432 | 3.840 to 5.023 | 0.2465 | 17.98 | 119.1 | 0.000000000000036 | **** |
| SomaFRCaMPi vs. SomaXCaMP-R | 3.391 | 2.776 to 4.006 | 0.2569 | 13.20 | 138.1 | 0.000000000000040 | **** |
| SomaFRCaMPi vs. SomajRGECO1a | 2.273 | 1.462 to 3.083 | 0.3398 | 6.689 | 211.1 | 0.000000000595164 | **** |
|  |  |  |  |  |  |  |  |
| **160** |  |  |  |  |  |  |  |
| SomaFRCaMPi vs. SomaK-GECO1 | 4.978 | 4.363 to 5.594 | 0.2559 | 19.45 | 96.77 | 0.000000000000008 | **** |
| SomaFRCaMPi vs. SomaXCaMP-R | 3.520 | 2.797 to 4.243 | 0.3023 | 11.64 | 145.1 | <0.000000000000001 | **** |
| SomaFRCaMPi vs. SomajRGECO1a | 2.511 | 1.342 to 3.680 | 0.4881 | 5.145 | 127.5 | 0.000002939429420 | **** |

**Descriptive Statistics on raw data for Peak SNR comparison (Fig 3M)**

|  | **SomaK-GECO1** | | | | | | |
| --- | --- | --- | --- | --- | --- | --- | --- |
| **# of Pulses** | **Mean** | **SEM** | **SD** | **N** | **Median** | **Upper limit (Q3)** | **Lower limit (Q1)** |
| 1 | 14.797929 | 1.1824409 | 7.8434254 | 44 | 12.41048 | 17.747243 | 9.613229 |
| 2 | 12.235949 | 0.9502015 | 6.1580093 | 42 | 10.77983 | 12.571785 | 9.3682818 |
| 3 | 14.190135 | 0.91938 | 5.6674392 | 38 | 12.078555 | 16.552053 | 10.279775 |
| 5 | 16.211204 | 1.1179445 | 7.4993997 | 45 | 14.25502 | 19.470365 | 11.114245 |
| 10 | 15.136815 | 1.5064416 | 9.8783981 | 43 | 12.13554 | 16.08605 | 9.11991 |
| 20 | 15.690031 | 0.998919 | 6.7749984 | 46 | 14.28971 | 18.250915 | 10.816595 |
| 40 | 16.300392 | 0.8581135 | 6.3058208 | 54 | 14.970055 | 18.954508 | 11.848688 |
| 80 | 18.396816 | 1.0503645 | 7.7897114 | 55 | 17.26734 | 22.1341 | 13.64953 |
| 160 | 22.354151 | 2.0010052 | 13.863371 | 48 | 19.179925 | 23.503675 | 14.350665 |

|  | **SomaXCaMP-R** | | | | | | |
| --- | --- | --- | --- | --- | --- | --- | --- |
| **# of Pulses** | **Mean** | **SEM** | **SD** | **N** | **Median** | **Upper limit (Q3)** | **Lower limit (Q1)** |
| 1 | 18.079296 | 1.0586089 | 7.6337376 | 52 | 16.83662 | 22.899125 | 12.584048 |
| 2 | 19.388317 | 0.8163003 | 6.2701217 | 59 | 18.71434 | 21.63091 | 15.21633 |
| 3 | 19.440044 | 0.8221844 | 6.4214656 | 61 | 18.80597 | 23.414735 | 14.90294 |
| 5 | 24.44208 | 0.978766 | 7.5180443 | 59 | 24.33239 | 29.4241 | 18.02059 |
| 10 | 26.450538 | 1.1132508 | 8.4048596 | 57 | 25.26086 | 30.02989 | 21.848275 |
| 20 | 25.626498 | 1.3140319 | 9.4756186 | 52 | 24.6774 | 31.571328 | 17.66339 |
| 40 | 25.020944 | 1.564205 | 10.493006 | 45 | 21.10015 | 33.160015 | 16.589905 |
| 80 | 29.987394 | 2.2143547 | 15.813655 | 51 | 23.33113 | 41.13125 | 17.9325 |
| 160 | 35.291161 | 3.020957 | 17.615055 | 34 | 27.603185 | 49.218038 | 23.045943 |

|  | **SomajRGECO1a** | | | | | | |
| --- | --- | --- | --- | --- | --- | --- | --- |
| **# of Pulses** | **Mean** | **SEM** | **SD** | **N** | **Median** | **Upper limit (Q3)** | **Lower limit (Q1)** |
| 1 | 21.787691 | 1.2656584 | 10.589258 | 70 | 21.01563 | 29.493465 | 13.959738 |
| 2 | 24.070985 | 1.3572903 | 9.7875593 | 52 | 20.22008 | 29.56432 | 17.557273 |
| 3 | 20.781851 | 1.0815115 | 8.5158302 | 62 | 19.136985 | 23.143518 | 14.960475 |
| 5 | 28.349856 | 1.5025825 | 13.771396 | 84 | 25.170295 | 31.588185 | 18.25609 |
| 10 | 29.628837 | 1.5073168 | 14.059316 | 87 | 26.93598 | 34.56834 | 20.28446 |
| 20 | 30.037032 | 1.8278574 | 16.652573 | 83 | 26.12635 | 34.39687 | 20.03897 |
| 40 | 31.858265 | 2.1670579 | 19.261232 | 79 | 27.66451 | 34.26007 | 20.79044 |
| 80 | 37.121082 | 2.2529451 | 20.525306 | 83 | 30.81732 | 42.38875 | 24.34321 |
| 160 | 47.237898 | 3.82174 | 27.023782 | 50 | 42.05369 | 56.438675 | 29.321848 |

|  | **SomaFRCaMPi** | | | | | | |
| --- | --- | --- | --- | --- | --- | --- | --- |
| **# of Pulses** | **Mean** | **SEM** | **SD** | **N** | **Median** | **Upper limit (Q3)** | **Lower limit (Q1)** |
| 1 | 41.852062 | 1.8154179 | 17.97172 | 98 | 39.589785 | 50.620985 | 28.841393 |
| 2 | 48.5024 | 1.8999716 | 20.726243 | 119 | 45.08013 | 64.80341 | 31.38408 |
| 3 | 49.346348 | 1.8448373 | 20.708247 | 126 | 46.340835 | 60.603378 | 34.933863 |
| 5 | 62.89153 | 2.3011702 | 26.136269 | 129 | 59.2932 | 76.4537 | 43.11573 |
| 10 | 74.793513 | 3.3201591 | 35.449598 | 114 | 67.496145 | 88.88261 | 51.325233 |
| 20 | 79.433601 | 3.3555098 | 37.365375 | 124 | 72.166445 | 96.86475 | 51.339125 |
| 40 | 89.443848 | 3.8730208 | 43.301689 | 125 | 78.19903 | 113.6496 | 58.4107 |
| 80 | 100.66152 | 3.9586851 | 44.43613 | 126 | 92.14004 | 121.28023 | 68.170733 |
| 160 | 114.09662 | 5.6112922 | 52.338649 | 87 | 102.0725 | 139.0065 | 80.0987 |

**Two-way ANOVA analysis for Peak SNR comparison (Fig 3M)**

| **Mixed-effects model (REML)** | **Matching: Stacked** |  |  |  |  |
| --- | --- | --- | --- | --- | --- |
| Assume sphericity? | No |  |  |  |  |
| Alpha | 0.05 |  |  |  |  |
|  |  |  |  |  |  |
| **Fixed effects (type III)** | **P value** | **P value summary** | **Statistically significant (P < 0.05)?** | **F (DFn, DFd)** | **Geisser-Greenhouse's epsilon** |
| # of Pulses | <0.000000000000001 | **** | Yes | F (2.545, 696.1) = 99.39 | 0.3182 |
| Red GECI | <0.000000000000001 | **** | Yes | F (3, 359) = 185.2 |  |
| # of Pulses x Red GECI | <0.000000000000001 | **** | Yes | F (24, 2188) = 36.97 |  |
|  |  |  |  |  |  |
| **Random effects** | **SD** | **Variance** |  |  |  |
| Subject | 18.83 | 354.5 |  |  |  |
| Residual | 15.34 | 235.4 |  |  |  |
|  |  |  |  |  |  |
| **Was the matching effective?** |  |  |  |  |  |
| Chi-square, df | 1457, 1 |  |  |  |  |
| P value | <0.000000000000001 |  |  |  |  |
| P value summary | **** |  |  |  |  |
| Is there significant matching (P < 0.05)? | Yes |  |  |  |  |
|  |  |  |  |  |  |
| **Data summary** |  |  |  |  |  |
| Number of columns (# of Pulses) | 4 |  |  |  |  |
| Number of rows (Red GECI) | 9 |  |  |  |  |
| Number of subjects (Subject) | 363 |  |  |  |  |
| Number of missing values | 684 |  |  |  |  |

**Dunnett post-hoc analysis for Peak SNR comparison (Fig 3M)**

|  | **Mean Diff.** | **95.00% CI of diff.** | **SE of diff.** | **q** | **DF** | **Adjusted P Value** | **Summary** |
| --- | --- | --- | --- | --- | --- | --- | --- |
| **# of Pulses:** |  |  |  |  |  |  |  |
| **1** |  |  |  |  |  |  |  |
| SomaFRCaMPi vs. SomaK-GECO1 | 27.05 | 21.86 to 32.25 | 2.167 | 12.49 | 139.9 | 0.000000000000023 | **** |
| SomaFRCaMPi vs. SomaXCaMP-R | 23.77 | 18.74 to 28.81 | 2.102 | 11.31 | 142.8 | <0.000000000000001 | **** |
| SomaFRCaMPi vs. SomajRGECO1a | 20.06 | 14.77 to 25.36 | 2.213 | 9.066 | 160.8 | 0.000000000000002 | **** |
|  |  |  |  |  |  |  |  |
| **2** |  |  |  |  |  |  |  |
| SomaFRCaMPi vs. SomaK-GECO1 | 36.27 | 31.17 to 41.36 | 2.124 | 17.07 | 156.3 | <0.000000000000001 | **** |
| SomaFRCaMPi vs. SomaXCaMP-R | 29.11 | 24.15 to 34.08 | 2.068 | 14.08 | 154.8 | 0.000000000000009 | **** |
| SomaFRCaMPi vs. SomajRGECO1a | 24.43 | 18.83 to 30.03 | 2.335 | 10.46 | 168.0 | 0.000000000000003 | **** |
|  |  |  |  |  |  |  |  |
| **3** |  |  |  |  |  |  |  |
| SomaFRCaMPi vs. SomaK-GECO1 | 35.16 | 30.21 to 40.10 | 2.061 | 17.06 | 161.2 | 0.000000000000012 | **** |
| SomaFRCaMPi vs. SomaXCaMP-R | 29.91 | 25.06 to 34.75 | 2.020 | 14.81 | 165.9 | <0.000000000000001 | **** |
| SomaFRCaMPi vs. SomajRGECO1a | 28.56 | 23.44 to 33.69 | 2.138 | 13.36 | 181.7 | <0.000000000000001 | **** |
|  |  |  |  |  |  |  |  |
| **5** |  |  |  |  |  |  |  |
| SomaFRCaMPi vs. SomaK-GECO1 | 46.68 | 40.55 to 52.81 | 2.558 | 18.25 | 168.3 | <0.000000000000001 | **** |
| SomaFRCaMPi vs. SomaXCaMP-R | 38.45 | 32.46 to 44.44 | 2.501 | 15.38 | 166.5 | <0.000000000000001 | **** |
| SomaFRCaMPi vs. SomajRGECO1a | 34.54 | 27.97 to 41.12 | 2.748 | 12.57 | 203.4 | 0.000000000000077 | **** |
|  |  |  |  |  |  |  |  |
| **10** |  |  |  |  |  |  |  |
| SomaFRCaMPi vs. SomaK-GECO1 | 59.66 | 50.92 to 68.39 | 3.646 | 16.36 | 147.5 | <0.000000000000001 | **** |
| SomaFRCaMPi vs. SomaXCaMP-R | 48.34 | 39.94 to 56.74 | 3.502 | 13.81 | 136.4 | 0.000000000000016 | **** |
| SomaFRCaMPi vs. SomajRGECO1a | 45.16 | 36.43 to 53.90 | 3.646 | 12.39 | 155.7 | <0.000000000000001 | **** |
|  |  |  |  |  |  |  |  |
| **20** |  |  |  |  |  |  |  |
| SomaFRCaMPi vs. SomaK-GECO1 | 63.74 | 55.34 to 72.15 | 3.501 | 18.21 | 142.7 | 0.000000000000044 | **** |
| SomaFRCaMPi vs. SomaXCaMP-R | 53.81 | 45.17 to 62.45 | 3.604 | 14.93 | 154.8 | <0.000000000000001 | **** |
| SomaFRCaMPi vs. SomajRGECO1a | 49.40 | 40.25 to 58.54 | 3.821 | 12.93 | 182.7 | <0.000000000000001 | **** |
|  |  |  |  |  |  |  |  |
| **40** |  |  |  |  |  |  |  |
| SomaFRCaMPi vs. SomaK-GECO1 | 73.14 | 63.62 to 82.67 | 3.967 | 18.44 | 135.7 | 0.000000000000045 | **** |
| SomaFRCaMPi vs. SomaXCaMP-R | 64.42 | 54.41 to 74.44 | 4.177 | 15.42 | 156.1 | <0.000000000000001 | **** |
| SomaFRCaMPi vs. SomajRGECO1a | 57.59 | 46.96 to 68.21 | 4.438 | 12.98 | 185.0 | 0.000000000000057 | **** |
|  |  |  |  |  |  |  |  |
| **80** |  |  |  |  |  |  |  |
| SomaFRCaMPi vs. SomaK-GECO1 | 82.26 | 72.44 to 92.09 | 4.096 | 20.09 | 141.6 | 0.000000000000005 | **** |
| SomaFRCaMPi vs. SomaXCaMP-R | 70.67 | 59.81 to 81.53 | 4.536 | 15.58 | 173.1 | <0.000000000000001 | **** |
| SomaFRCaMPi vs. SomajRGECO1a | 63.54 | 52.64 to 74.44 | 4.555 | 13.95 | 188.9 | 0.000000000000003 | **** |
|  |  |  |  |  |  |  |  |
| **160** |  |  |  |  |  |  |  |
| SomaFRCaMPi vs. SomaK-GECO1 | 91.74 | 77.40 to 106.1 | 5.957 | 15.40 | 106.1 | <0.000000000000001 | **** |
| SomaFRCaMPi vs. SomaXCaMP-R | 78.81 | 63.48 to 94.13 | 6.373 | 12.37 | 117.4 | <0.000000000000001 | **** |
| SomaFRCaMPi vs. SomajRGECO1a | 66.86 | 50.56 to 83.15 | 6.789 | 9.848 | 133.8 | <0.000000000000001 | **** |
