# Supplementary material for: A sensitive soma-localized red fluorescent calcium indicator for in vivo imaging of neuronal populations at single-cell resolution
Source: PLoS Biol. 2025 Apr 29;23(4):e3003048. doi: 10.1371/journal.pbio.3003048 (PMC12040222; doi:10.1371/journal.pbio.3003048)
Supplement: S6 Table — (DOCX) [file pbio.3003048.s022.docx]

**S6 Table. Statistics on FRCaMPi and SomaFRCaMPi characterization in wide-field one-photon cortical imaging of awake mice**

**Descriptive Statistics on raw data for number of ROIs identified per FOV (Fig 7D)**

|  | FRCaMPi S1 | SomaFRCaMPi S1 | FRCaMPi V1 | SomaFRCaMPi V1 |
| --- | --- | --- | --- | --- |
| Number of values | 4 | 6 | 3 | 5 |
|  |  |  |  |  |
| Minimum | 23.00 | 97.00 | 32.00 | 236.0 |
| 25% Percentile | 26.75 | 108.3 | 32.00 | 239.0 |
| Median | 43.00 | 141.5 | 96.00 | 276.0 |
| 75% Percentile | 51.00 | 319.3 | 435.0 | 449.5 |
| Maximum | 52.00 | 338.0 | 435.0 | 468.0 |
| Range | 29.00 | 241.0 | 403.0 | 232.0 |
|  |  |  |  |  |
| 10% Percentile | 23.00 | 97.00 | 32.00 | 236.0 |
| 90% Percentile | 52.00 | 338.0 | 435.0 | 468.0 |
|  |  |  |  |  |
| 95% CI of median |  |  |  |  |
| Actual confidence level | 87.50% | 96.88% | 75.00% | 93.75% |
| Lower confidence limit | 23.00 | 97.00 | 32.00 | 236.0 |
| Upper confidence limit | 52.00 | 338.0 | 435.0 | 468.0 |
|  |  |  |  |  |
| Mean | 40.25 | 190.5 | 187.7 | 330.6 |
| Std. Deviation | 12.92 | 106.4 | 216.6 | 110.4 |
| Std. Error of Mean | 6.460 | 43.42 | 125.0 | 49.37 |
|  |  |  |  |  |
| Lower 95% CI of mean | 19.69 | 78.89 | -350.3 | 193.5 |
| Upper 95% CI of mean | 60.81 | 302.1 | 725.7 | 467.7 |

**Descriptive Statistics on raw data for baseline brightness comparison between FRCaMPi and SomaFRCaMPi (Fig 7F)**

|  | FRCaMPi S1 | SomaFRCaMPi S1 | FRCaMPi V1 | SomaFRCaMPi V1 |
| --- | --- | --- | --- | --- |
| Number of values | 161 | 1143 | 563 | 1653 |
|  |  |  |  |  |
| Minimum | 458.0 | 436.1 | 544.7 | 369.2 |
| 25% Percentile | 697.7 | 565.8 | 1219 | 589.9 |
| Median | 833.6 | 642.7 | 1490 | 637.5 |
| 75% Percentile | 917.2 | 719.8 | 1600 | 685.4 |
| Maximum | 1018 | 900.9 | 1708 | 846.5 |
| Range | 559.5 | 464.9 | 1163 | 477.4 |
|  |  |  |  |  |
| 10% Percentile | 552.5 | 525.5 | 905.3 | 538.3 |
| 90% Percentile | 964.7 | 816.1 | 1669 | 762.9 |
|  |  |  |  |  |
| 95% CI of median |  |  |  |  |
| Actual confidence level | 95.99% | 95.00% | 95.70% | 95.00% |
| Lower confidence limit | 807.4 | 634.4 | 1475 | 632.6 |
| Upper confidence limit | 878.8 | 651.7 | 1510 | 642.6 |
|  |  |  |  |  |
| Mean | 801.3 | 653.6 | 1374 | 641.8 |
| Std. Deviation | 147.1 | 107.2 | 303.0 | 86.21 |
| Std. Error of Mean | 11.60 | 3.171 | 12.77 | 2.120 |
|  |  |  |  |  |
| Lower 95% CI of mean | 778.3 | 647.4 | 1349 | 637.7 |
| Upper 95% CI of mean | 824.2 | 659.9 | 1399 | 646.0 |

**Mann–Whitney U-test between baseline brightness of FRCaMPi and SomaFRCaMPi in S1 (Fig 7F)**

| P value | <0.000000000000001 |
| --- | --- |
| Exact or approximate P value? | Approximate |
| P value summary | **** |
| Significantly different (P < 0.05)? | Yes |
| One- or two-tailed P value? | Two-tailed |
| Sum of ranks in column A,B | 157263 , 693597 |
| Mann-Whitney U | 39801 |

**Mann–Whitney U-test between baseline brightness of FRCaMPi and SomaFRCaMPi in V1 (Fig 7F)**

| P value | <0.000000000000001 |
| --- | --- |
| Exact or approximate P value? | Approximate |
| P value summary | **** |
| Significantly different (P < 0.05)? | Yes |
| One- or two-tailed P value? | Two-tailed |
| Sum of ranks in column C,D | 1075625 , 1380811 |
| Mann-Whitney U | 13780 |

**Descriptive Statistics on raw data for Peak ΔF/F_0_ comparison between FRCaMPi and SomaFRCaMPi (Fig 7G)**

|  | FRCaMPi S1 | SomaFRCaMPi S1 | FRCaMPi V1 | SomaFRCaMPi V1 |
| --- | --- | --- | --- | --- |
| Number of values | 161 | 1143 | 563 | 1653 |
|  |  |  |  |  |
| Minimum | 0.2652 | 0.08953 | 0.04768 | 0.02292 |
| 25% Percentile | 0.4302 | 0.4053 | 0.5608 | 0.5176 |
| Median | 0.5892 | 0.5696 | 0.7542 | 1.510 |
| 75% Percentile | 1.560 | 2.050 | 1.091 | 2.271 |
| Maximum | 7.142 | 6.229 | 4.669 | 9.229 |
| Range | 6.876 | 6.139 | 4.621 | 9.207 |
|  |  |  |  |  |
| 10% Percentile | 0.3354 | 0.3194 | 0.4430 | 0.3840 |
| 90% Percentile | 3.314 | 2.969 | 1.485 | 3.004 |
|  |  |  |  |  |
| 95% CI of median |  |  |  |  |
| Actual confidence level | 95.99% | 95.00% | 95.70% | 95.00% |
| Lower confidence limit | 0.5142 | 0.5373 | 0.7064 | 1.452 |
| Upper confidence limit | 0.6347 | 0.6104 | 0.7859 | 1.577 |
|  |  |  |  |  |
| Mean | 1.189 | 1.239 | 0.9128 | 1.545 |
| Std. Deviation | 1.248 | 1.140 | 0.5812 | 1.076 |
| Std. Error of Mean | 0.09834 | 0.03371 | 0.02449 | 0.02647 |
|  |  |  |  |  |
| Lower 95% CI of mean | 0.9949 | 1.173 | 0.8647 | 1.493 |
| Upper 95% CI of mean | 1.383 | 1.305 | 0.9609 | 1.597 |

**Mann–Whitney U-test between Peak ΔF/F_0_ of FRCaMPi and SomaFRCaMPi in S1 (Fig 7G)**

| P value | 0.842128655659912 |
| --- | --- |
| Exact or approximate P value? | Approximate |
| P value summary | ns |
| Significantly different (P < 0.05)? | No |
| One- or two-tailed P value? | Two-tailed |
| Sum of ranks in column A,B | 105944 , 744916 |
| Mann-Whitney U | 91120 |

**Mann–Whitney U-test between Peak ΔF/F_0_ of FRCaMPi and SomaFRCaMPi in V1 (Fig 7G)**

| P value | <0.000000000000001 |
| --- | --- |
| Exact or approximate P value? | Approximate |
| P value summary | **** |
| Significantly different (P < 0.05)? | Yes |
| One- or two-tailed P value? | Two-tailed |
| Sum of ranks in column C,D | 497869 , 1958567 |
| Mann-Whitney U | 339103 |

**Descriptive Statistics on raw data for Peak SNR comparison between FRCaMPi and SomaFRCaMPi (Fig 7H)**

|  | FRCaMPi S1 | SomaFRCaMPi S1 | FRCaMPi V1 | SomaFRCaMPi V1 |
| --- | --- | --- | --- | --- |
| Number of values | 161 | 1143 | 563 | 1653 |
|  |  |  |  |  |
| Minimum | 5.139 | 4.953 | 5.117 | 5.689 |
| 25% Percentile | 8.366 | 9.121 | 7.537 | 9.991 |
| Median | 10.53 | 11.85 | 9.088 | 12.69 |
| 75% Percentile | 15.17 | 15.86 | 11.50 | 16.64 |
| Maximum | 57.25 | 48.31 | 32.07 | 78.18 |
| Range | 52.12 | 43.36 | 26.95 | 72.49 |
|  |  |  |  |  |
| 10% Percentile | 7.319 | 7.791 | 6.555 | 8.120 |
| 90% Percentile | 28.19 | 20.36 | 15.00 | 21.49 |
|  |  |  |  |  |
| 95% CI of median |  |  |  |  |
| Actual confidence level | 95.99% | 95.00% | 95.70% | 95.00% |
| Lower confidence limit | 10.00 | 11.49 | 8.925 | 12.31 |
| Upper confidence limit | 11.53 | 12.31 | 9.463 | 12.93 |
|  |  |  |  |  |
| Mean | 13.77 | 13.19 | 10.13 | 14.10 |
| Std. Deviation | 8.822 | 5.454 | 3.819 | 6.225 |
| Std. Error of Mean | 0.6953 | 0.1613 | 0.1610 | 0.1531 |
|  |  |  |  |  |
| Lower 95% CI of mean | 12.39 | 12.88 | 9.815 | 13.80 |
| Upper 95% CI of mean | 15.14 | 13.51 | 10.45 | 14.40 |

**Mann–Whitney U-test between Peak SNR of FRCaMPi and SomaFRCaMPi in S1 (Fig 7H)**

| P value | 0.031698660444950 |
| --- | --- |
| Exact or approximate P value? | Approximate |
| P value summary | * |
| Significantly different (P < 0.05)? | Yes |
| One- or two-tailed P value? | Two-tailed |
| Sum of ranks in column A,B | 95442, 755418 |
| Mann-Whitney U | 82401 |

**Mann–Whitney U-test between Peak SNR of FRCaMPi and SomaFRCaMPi in V1 (Fig 7H)**

| P value | <0.000000000000001 |
| --- | --- |
| Exact or approximate P value? | Approximate |
| P value summary | **** |
| Significantly different (P < 0.05)? | Yes |
| One- or two-tailed P value? | Two-tailed |
| Sum of ranks in column C,D | 398162 , 2058274 |
| Mann-Whitney U | 239396 |

**Descriptive Statistics on raw data for decay time comparison between FRCaMPi and SomaFRCaMPi (Fig 7I)**

|  | FRCaMPi S1 | SomaFRCaMPi S1 | FRCaMPi V1 | SomaFRCaMPi V1 |
| --- | --- | --- | --- | --- |
| Number of values | 56 | 595 | 343 | 1247 |
|  |  |  |  |  |
| Minimum | 0.3750 | 0.07500 | 0.1500 | 0.07500 |
| 25% Percentile | 0.5250 | 0.6000 | 0.5250 | 0.6000 |
| Median | 0.7500 | 0.7500 | 0.8250 | 0.7500 |
| 75% Percentile | 1.031 | 0.9750 | 1.125 | 1.050 |
| Maximum | 1.650 | 5.175 | 2.325 | 4.575 |
| Range | 1.275 | 5.100 | 2.175 | 4.500 |
|  |  |  |  |  |
| 10% Percentile | 0.4500 | 0.4500 | 0.4500 | 0.4500 |
| 90% Percentile | 1.275 | 1.275 | 1.500 | 1.350 |
|  |  |  |  |  |
| 95% CI of median |  |  |  |  |
| Actual confidence level | 95.60% | 95.10% | 96.00% | 95.00% |
| Lower confidence limit | 0.6750 | 0.7500 | 0.7500 | 0.7500 |
| Upper confidence limit | 0.8250 | 0.7500 | 0.9000 | 0.8250 |
|  |  |  |  |  |
| Mean | 0.8089 | 0.8195 | 0.8884 | 0.8557 |
| Std. Deviation | 0.3197 | 0.4065 | 0.4325 | 0.4041 |
| Std. Error of Mean | 0.04272 | 0.01666 | 0.02335 | 0.01144 |
|  |  |  |  |  |
| Lower 95% CI of mean | 0.7233 | 0.7867 | 0.8425 | 0.8332 |
| Upper 95% CI of mean | 0.8945 | 0.8522 | 0.9343 | 0.8781 |

**Mann–Whitney U-test between decay time of FRCaMPi and SomaFRCaMPi in S1 (Fig 7I)**

| P value | 0.957463218054911 |
| --- | --- |
| Exact or approximate P value? | Exact |
| P value summary | ns |
| Significantly different (P < 0.05)? | No |
| One- or two-tailed P value? | Two-tailed |
| Sum of ranks in column A,B | 18328 , 193898 |
| Mann-Whitney U | 16588 |

**Mann–Whitney U-test between decay time of FRCaMPi and SomaFRCaMPi in V1 (Fig 7I)**

| P value | 0.392886334404986 |
| --- | --- |
| Exact or approximate P value? | Approximate |
| P value summary | ns |
| Significantly different (P < 0.05)? | No |
| One- or two-tailed P value? | Two-tailed |
| Sum of ranks in column C,D | 279277 , 985568 |
| Mann-Whitney U | 207440 |

**Descriptive Statistics on raw data for spike rate comparison between FRCaMPi and SomaFRCaMPi (Fig 7J)**

|  | FRCaMPi S1 | SomaFRCaMPi S1 | FRCaMPi V1 | SomaFRCaMPi V1 |
| --- | --- | --- | --- | --- |
| Number of values | 195 | 1194 | 635 | 1773 |
|  |  |  |  |  |
| Minimum | 0.02667 | 0.02667 | 0.02667 | 0.02667 |
| 25% Percentile | 0.02667 | 0.02667 | 0.02667 | 0.02667 |
| Median | 0.02667 | 0.02667 | 0.02667 | 0.05333 |
| 75% Percentile | 0.05333 | 0.08 | 0.05333 | 0.08 |
| Maximum | 0.2133 | 0.4267 | 0.2667 | 0.48 |
| Range | 0.1867 | 0.4 | 0.24 | 0.4533 |
|  |  |  |  |  |
| 10% Percentile | 0.02667 | 0.02667 | 0.02667 | 0.02667 |
| 90% Percentile | 0.08 | 0.1333 | 0.08 | 0.16 |
|  |  |  |  |  |
| 95% CI of median | |  |  |  |
| Actual confidence level | 95.53% | 95.00% | 95.29% | 95.00% |
| Lower confidence limit | 0.02667 | 0.02667 | 0.02667 | 0.05333 |
| Upper confidence limit | 0.02667 | 0.05333 | 0.02667 | 0.05333 |
|  |  |  |  |  |
| Mean | 0.04444 | 0.05889 | 0.04934 | 0.07159 |
| Std. Deviation | 0.03516 | 0.05171 | 0.03618 | 0.06397 |
| Std. Error of Mean | 0.002518 | 0.001496 | 0.001436 | 0.001519 |
|  |  |  |  |  |
| Lower 95% CI of mean | 0.03948 | 0.05596 | 0.04652 | 0.06861 |
| Upper 95% CI of mean | 0.04941 | 0.06183 | 0.05216 | 0.07457 |

**Mann–Whitney U-test between spike rate of FRCaMPi and SomaFRCaMPi in S1 (Fig 7J)**

| P value | 0.000017567131268 |
| --- | --- |
| Exact or approximate P value? | Approximate |
| P value summary | **** |
| Significantly different (P < 0.05)? | Yes |
| One- or two-tailed P value? | Two-tailed |
| Sum of ranks in column A,B | 115283 , 850072 |
| Mann-Whitney U | 96173 |

**Mann–Whitney U-test between spike rate of FRCaMPi and SomaFRCaMPi in V1 (Fig 7J)**

| P value | <0.000000000000001 |
| --- | --- |
| Exact or approximate P value? | Approximate |
| P value summary | **** |
| Significantly different (P < 0.05)? | Yes |
| One- or two-tailed P value? | Two-tailed |
| Sum of ranks in column C,D | 544055 , 2356382 |
| Mann-Whitney U | 342125 |

**Descriptive Statistics on raw data for comparison of correlation coefficient between FRCaMPi and SomaFRCaMPi cell pairs (Fig 7K-M)**

|  | FRCaMPi S1 | SomaFRCaMPi S1 | FRCaMPi V1 | SomaFRCaMPi V1 |
| --- | --- | --- | --- | --- |
| Number of values | 3410 | 136576 | 99451 | 296784 |
|  |  |  |  |  |
| Minimum | 0.6849 | 0.3003 | 0.8028 | 0.5282 |
| 25% Percentile | 0.8855 | 0.7757 | 0.9428 | 0.8396 |
| Median | 0.9275 | 0.8760 | 0.9655 | 0.9047 |
| 75% Percentile | 0.9610 | 0.9409 | 0.9829 | 0.9511 |
| Maximum | 0.9999 | 0.9998 | 1.000 | 0.9999 |
| Range | 0.3150 | 0.6995 | 0.1972 | 0.4718 |
|  |  |  |  |  |
| 10% Percentile | 0.8188 | 0.6471 | 0.9210 | 0.7562 |
| 90% Percentile | 0.9829 | 0.9735 | 0.9926 | 0.9756 |
|  |  |  |  |  |
| 95% CI of median |  |  |  |  |
| Actual confidence level | 95.00% | 95.00% | 95.00% | 95.00% |
| Lower confidence limit | 0.9254 | 0.8751 | 0.9653 | 0.9043 |
| Upper confidence limit | 0.9304 | 0.8769 | 0.9658 | 0.9051 |
|  |  |  |  |  |
| Mean | 0.9149 | 0.8411 | 0.9602 | 0.8844 |
| Std. Deviation | 0.06206 | 0.1278 | 0.02859 | 0.08589 |
| Std. Error of Mean | 0.001063 | 0.0003459 | 9.066e-005 | 0.0001577 |
|  |  |  |  |  |
| Lower 95% CI of mean | 0.9128 | 0.8404 | 0.9600 | 0.8841 |
| Upper 95% CI of mean | 0.9169 | 0.8418 | 0.9604 | 0.8847 |

**Mann–Whitney U-test between correlation coefficient of FRCaMPi and SomaFRCaMPi cell pairs in S1 (Fig 7K)**

| P value | <0.000000000000001 |
| --- | --- |
| Exact or approximate P value? | Approximate |
| P value summary | **** |
| Significantly different (P < 0.05)? | Yes |
| One- or two-tailed P value? | Two-tailed |
| Sum of ranks in column A,B | 318734292 , 9479375799 |
| Mann-Whitney U | 152805623 |

**Mann–Whitney U-test between correlation coefficient of FRCaMPi and SomaFRCaMPi cell pairs in V1 (Fig 7K)**

| P value | <0.000000000000001 |
| --- | --- |
| Exact or approximate P value? | Approximate |
| P value summary | **** |
| Significantly different (P < 0.05)? | Yes |
| One- or two-tailed P value? | Two-tailed |
| Sum of ranks in column C,D | 28749623094, 49751662636 |
| Mann-Whitney U | 5711142916 |
