# Supplementary material for: A sensitive soma-localized red fluorescent calcium indicator for in vivo imaging of neuronal populations at single-cell resolution
Source: PLoS Biol. 2025 Apr 29;23(4):e3003048. doi: 10.1371/journal.pbio.3003048 (PMC12040222; doi:10.1371/journal.pbio.3003048)
Supplement: S1 Text — (DOCX) [file pbio.3003048.s023.docx]

**S1 Text. Challenges and strategies in reporting single neuronal activities using wide-field imaging**

Wide-field microscopy is a cost-effective approach that enables capturing the somatic activity of thousands of neurons across brain regions of millimeter-scale with video-rate data^1-4^. However, extraction of somatic signals is challenging in widefield imaging due to scattering tissue and fluorescence from neuropils and neurons out of the focal plane. Therefore, strategies such as patterned illumination techniques^5,6^ and layer-specific labeling^1-4,6,7^ for sparser signals were often adapted in parallel to facilitate signal extraction at the single-cell bodies and reduce artificial signal contamination for imaging large neuronal populations in wide-field setups. Nevertheless, a more accessible and straightforward strategy compared to these technical circumventions could be the somatic localization of the GECI. This approach should directly reduce neuropil fluorescence in the background without biasing somatic signals and potentially demonstrates more efficient and accurate signal extraction in spite of high neuronal labeling density during neuronal activation. Further engineering of soma-localized GCaMPs by EE-RR motif has been shown to improve the number and accuracy of somatic calcium signals extracted from wide-field imaging datasets. However, the performance of the indicator can be substantially compromised by spilling in neuropil expression^8^. An indicator with restricted soma-localization paired with a strong sensitivity would, in principle, directly reduce neuropil fluorescence in the background and facilitate somatic signal detection in spite of a high expression density. Previous attempts in wide-field imaging that reported single neuronal activities were dominated by green indicators such as GCaMPs^1-4^. Few were reported for red^6^, plausibly due to their lower SNR *in vivo*.

**References**

1 Fan, J. *et al.* Video-rate imaging of biological dynamics at centimetre scale and micrometre resolution. *Nature Photonics* **13**, 809-816 (2019). <https://doi.org:10.1038/s41566-019-0474-7>

2 Zhang, Y. *et al.* Rapid detection of neurons in widefield calcium imaging datasets after training with synthetic data. *Nature Methods* **20**, 747-754 (2023). <https://doi.org:10.1038/s41592-023-01838-7>

3 Kim, T. H. *et al.* Long-Term Optical Access to an Estimated One Million Neurons in the Live Mouse Cortex. *Cell Reports* **17**, 3385-3394 (2016). <https://doi.org:https://doi.org/10.1016/j.celrep.2016.12.004>

4 Zhang, Y. *et al.* A miniaturized mesoscope for the large-scale single-neuron-resolved imaging of neuronal activity in freely behaving mice. *Nature Biomedical Engineering* **8**, 754-774 (2024). <https://doi.org:10.1038/s41551-024-01226-2>

5 Szabo, V., Ventalon, C., De Sars, V., Bradley, J. & Emiliani, V. Spatially Selective Holographic Photoactivation and Functional Fluorescence Imaging in Freely Behaving Mice with a Fiberscope. *Neuron* **84**, 1157-1169 (2014). <https://doi.org:https://doi.org/10.1016/j.neuron.2014.11.005>

6 Zhang, J. *et al.* A one-photon endoscope for simultaneous patterned optogenetic stimulation and calcium imaging in freely behaving mice. *Nature Biomedical Engineering* **7**, 499-510 (2023). <https://doi.org:10.1038/s41551-022-00920-3>

7 Manley, J. *et al.* Simultaneous, cortex-wide dynamics of up to 1 million neurons reveal unbounded scaling of dimensionality with neuron number. *Neuron* **112**, 1694-1709.e1695 (2024). <https://doi.org:https://doi.org/10.1016/j.neuron.2024.02.011>

8 Grødem, S. *et al.* An updated suite of viral vectors for in vivo calcium imaging using intracerebral and retro-orbital injections in male mice. *Nature Communications* **14**, 608 (2023). <https://doi.org:10.1038/s41467-023-36324-3>
