# Supplementary material for: A sensitive soma-localized red fluorescent calcium indicator for in vivo imaging of neuronal populations at single-cell resolution
Source: PLoS Biol. 2025 Apr 29;23(4):e3003048. doi: 10.1371/journal.pbio.3003048 (PMC12040222; doi:10.1371/journal.pbio.3003048)
Supplement: Attachment — Submitted filename: Response to Reviewers 25.2.28.docx [file pbio.3003048.s028.docx]

Reviewer remarks:

Reviewer #1*: The authors have addressed all my concerns. The addition of the GCaMP experiments have significantly improved the message of the paper and it is suitable for publication in Plos biology in its current form*.

We sincerely appreciate the Reviewer’s insightful feedback and positive evaluation of our work. We are deeply grateful for their support of our manuscript’s publication in PLOS Biology.

*Reviewer #2: In response to the previous round of reviews, the authors have added experiments to compare somaFRCaMPi with soma-localized GCaMP8s using 2P imaging in mouse brain, and shown that they perform similarly. In addition to other edits they have also added discussion about potential photoswitching of their sensor that may limit its utility. Although the authors have still not experimentally addressed the important potential limitations of this type of sensor (photoswitching, incomplete maturation in vivo) or experimentally demonstrated any application highlighting why someone would use a red GECI (multiplexed functional imaging, deep tissue imaging, combination with channelrhodopsin), which significantly limits the impact of this work, I will still recommend publication because they demonstrate some situations where their sensor does work and it appears to be the current best-in-class red FP-based GECI.*

We sincerely thank Reviewer for their constructive feedback and insightful comments. The concern regarding photoswitching is a key challenge in the development of red fluorescent protein-based probes, and we fully acknowledge its importance. In response, we commit to thoroughly investigating the photoswitching behavior of our probe in future studies, including a detailed characterization of its photoswitching to elucidate the underlying genetic component and mechanism. We are thankful for the reviewer’s approval of our work for publication in PLOS Biology.
